# On the Design of Effective Water‐Soluble Actinide‐Masking Ligands Through Ligand Structure Modulation

**DOI:** 10.1002/advs.202512292

**Published:** 2025-08-21

**Authors:** Bin Li, Yu Kang, Ziyi Zhang, Ludi Wang, Haoyu Li, Yuxiao Guo, Guo Wang, Li Wang, Xiaoyan Tang, Chao Xu

**Affiliations:** ^1^ Institute of Nuclear and New Energy Technology Tsinghua University Haidian District Beijing 100084 China; ^2^ Department of Chemistry Capital Normal University Haidian District Beijing 100048 China; ^3^ Beijing National Laboratory for Molecular Sciences Key Laboratory of Polymer Chemistry and Physics of Ministry of Education Centre for Soft Matter Science and Engineering College of Chemistry and Molecular Engineering Peking University Haidian District Beijing 100871 China

**Keywords:** f‐Block element, Ligand design, Liquid‐liquid separation, Selective coordination, Water‐soluble

## Abstract

Water‐soluble actinide‐masking ligands are fundamentally important for achieving efficient lanthanide/actinide separation and for the development of water‐soluble f‐block complexes for bioimaging and radiopharmaceutical applications. However, the underlying design principles remain largely elusive, particularly in achieving a fine balance between ligand water solubility and metal affinity/selectivity. In this study, it is demonstrated that for the well‐established phenanthroline diimine ligand framework, topological modifications can preserve water solubility but introduce significant rotational energy barriers. These barriers, in turn, diminish both the metal‐binding affinity and selectivity. Conversely, non‐coordinating substituents play an unexpected role in modulating water solubility. Specifically, the incorporation of methylthio‐flanking groups is found to significantly impair the ligand's aqueous solubility. A combination of solution‐ and solid‐state coordination studies is employed to elucidate how structural modifications influence ligand‐metal interactions. Additionally, DFT calculations provided molecular‐level insights into the relationship between chemical structure, water solubility, and coordination behavior. This work offers valuable design guidelines for the development of hydrophilic ligands, with implications for selective f‐block element separation and the formulation of stable, water‐soluble f‐block complexes.

## Graphical abstract

Ligand topology and non‐coordination atoms are proved to be important for the design of effective water‐soluble actinide masking agents.

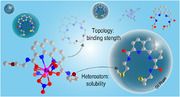

## 1 Introduction

The global energy landscape, driven by surging electricity demands in fields such as electric vehicles and data centers, has catalyzed a nuclear power resurgence.^[^ ^1^ ^]^ As reported by the World Nuclear Association (2024), 440 operational reactors currently supply 10% of global electricity and 25% of low‐carbon power, with nations accelerating reactor life‐extension programs and developing of advanced reactors.^[^ ^2^ ^]^ However, the large amount (about tens of thousands of tons per year) of spent nuclear fuel (SNF) generated during the operation of nuclear reactors is the hanging Sword of Damocles, becoming a major problem that urgently needs to be solved on the path of the sustainable development of nuclear energy.^[^ ^2^, ^3^ ^]^ The resultant SNF, a complex cocktail of radionuclides spanning microsecond to billion‐year half‐lives (e.g., 24000 years of Pu‐239 vs 30 years of Cs‐137), poses critical sustainability challenges.^[^ ^2^, ^3^, ^4^ ^]^ The inability to achieve precise radionuclide partitioning remains a fundamental barrier to close the nuclear fuel cycle.^[^ ^2^, ^5^ ^]^ As one of the most widely applied industrial processes, the PUREX process successfully recovers > 95% uranium/plutonium in SNF and makes it possible for SNF to enter the “uranium‐plutonium cycle”.^[^ ^6^ ^]^ However, the remaining raffinate, namely high‐level liquid waste (HLLW) presents formidable hazards due to its high acidity and high radioactive toxicity. The minor actinides (MAs, such as Np, Am, Cm) and lanthanides form a deadly combination. The former dominates the million‐year radiotoxicity profile of HLLW, while the latter severely inhibits the neutron transmutation efficiency of Ans due to their high neutron cross sections, which hinders the utilization of the partitioning and transmutation process to convert Ans into short‐lived radionuclides.^[^ ^2^, ^3^, ^7^ ^]^ Moreover, Lns tend to segregate into isolated phase and induce fuel pellet heterogeneity during fuel irradiation, resulting in uneven heat distribution in the fuel matrix.^[^ ^7f^ ^]^ Therefore, the separation of Ans/Lns is the core in the “detoxification” chain of SNF. The development of advanced processes (such as TRUEX, DIAMEX, TALSPEAK, and SANEX/*i*‐SANEX) has been spurred aiming to achieve the efficient separation of Ans/Lns. Yet the industrialization of above‐mentioned processes remains constrained by the great challenge of efficient separation due to the extremely similar physical and chemical properties of Ans/Lns (similar charge structures, ionic radii, same charge number, and similar thermodynamic properties, etc.).^[^ ^2^, ^3^, ^5^, ^6^, ^8^ ^]^

Current strategies of Ans/Lns separation mainly bifurcate into oxidation‐state control and coordination chemistry‐based liquid‐liquid separation (CCBLLS).^[^ ^7^, ^9^ ^]^ The former approach, although theoretically efficient, is limited by operational complexity.^[^ ^6^, ^9^ ^]^ It typically requires large amounts of oxidants and involves redox‐unstable compounds. Additionally, the use of corrosive reagents poses compatibility risks with industrial equipment. In contrast, the latter strategy leverages the stronger covalency of the 5f orbitals of An(III) ions with soft donor atoms such as nitrogen and sulfur based on Pearson's hard‐soft acid‐base (HSAB) theory.^[^ ^3^, ^5^ ^]^ This method is more suitable for industrial‐scale applications. Two kinds of ligands were mostly explored in the literature based on their application environments: lipophilic and hydrophilic ligands.^[^ ^2^, ^3^ ^]^ Earlier studies focused on lipophilic ligands, with **TODGA**, **Cyanex 301**, and **CyMe4‐BTPhen** being among the most successful examples (Scheme S1, Supporting Information).^[^ ^3^, ^5^ ^]^ However, the presence of multiple cations with nearly identical properties, combined with the need for simplified processes to achieve both group separation of Ln(III)/An(III) and efficient separation of neighboring Ln(III)/Ln(III) or An(III)/An(III), presents significant challenges.^[^ ^2^, ^5^, ^6^, ^10^ ^]^

To address these issues, hydrophilic ligands have been developed which are designed for use in the aqueous phase to selectively retain or strip specific metal cations.^[^ ^2^, ^10^ ^]^ When paired with lipophilic ligands that exhibit complementary selectivity, a “tug‐of‐war” strategy emerges – offering the potential to overcome the challenges of both intergroup and intragroup separations of lanthanides and actinides.^[^ ^7^, ^9^, ^11^ ^]^ The key design principles for effective hydrophilic ligands are high metal selectivity and sufficient water solubility.^[^ ^2^, ^3^, ^6^, ^10^ ^]^ Metal selectivity can be achieved by adopting successful features from lipophilic ligands, such as ligand preorganization and the use of appropriate hard/soft donor atoms.^[^ ^7e,f^ ^]^ Water solubility is typically ensured by incorporating functional groups like sulfonates or hydroxyls (Scheme S2, Supporting Information).^[^ ^2^, ^3^ ^]^ However, significant challenges still remain for the design and application of hydrophilic ligands, with key issues including: (1) complex syntheses with unstable intermediates and costly purification; (2) strong solvation competition from water molecules that reduces metal‐ion selectivity; and (3) poor acid tolerance, as most ligands lose performance above 1.0 M HNO_3_. Balancing solubility and selectivity, particularly under highly acidic and competitive conditions, remains a major obstacle.^[^ ^2^, ^10^ ^]^

Recently, we have reported a series of phenanthroline diimine ligands featuring tetradentate N, O‐coordination sites for the efficient separation of Eu(III)/Am(III) and Cm(III)/Am(III) under highly acidic conditions.^[^ ^2^, ^7^, ^12^ ^]^ These ligands achieved high separation factors of ≈300 for Eu(III)/Am(III) and 10 for Cm(III)/Am(III) in nitric acid solutions exceeding 1 M. The impressive selectivity was attributed to the preorganized ligand architecture and the hydrogen‐bonding capability of the imine groups. Additionally, we found that the flanking terminal groups play a critical role in determining both the water solubility and acid resistance of the ligands. Efforts to enhance selectivity by tuning the electronic properties of the ligands‐such as introducing bromine substituents‐resulted in a complete loss of water solubility,^[^ ^12e^ ^]^ even when using positively charged, amine‐containing analogues (which previously showed solubility above 0.2 m in 0.25 m HNO_3_).^[^ ^12b^ ^]^ These results suggest that the previously reported ligands lie at a finely tuned balance point between metal selectivity and aqueous solubility. To further improve ligand performance, more detailed molecular‐level insights are needed to elucidate the structural features governing both selectivity and solubility. In the current work, three ligands based on the phenanthroline diimine (**Phen‐2DI**) architecture were designed and synthesized. Direct comparisons between 1,2‐bis(2‐aminoethoxy)ethane (**Phen‐2DICy**) and 2‐(methylthio)ethylamine group (**Phen‐2DIC2SMe**) substituted **Phen‐2DI** with the 2‐methoxyethylamine modified ligands (**Phen‐2DIC2OMe**) were conducted to show the ligand topology and heteroatom effects on the overall selectivity and water solubility. With respect to **Phen‐2DIC2OMe**, **Phen‐2DICy** preserved the water solubility while lost the selectivity toward both the Eu(III)/Am(III) and Cm(III)/Am(III). While the introduction of non‐coordinating sulfur crippled the ligand water solubility. Multiple titrations methods were used to show the solution coordination behaviors and DFT calculations were performed to reveal the underlying mechanisms to shed more light on the further rational design of highly efficient hydrophilic f‐block coordination ligands. Critically, although preorganization and imine hydrogen‐bonding have been established to be critical for ligand selectivity,^[^ ^7^, ^9^, ^11^, ^12^ ^]^ this work further demonstrates that terminal groups act as critical modulators of metal affinity through topological and electronic effects which can override core‐derived selectivity‐a design principle previously underestimated in hydrophilic ligand engineering. Overall, this study provides important design principles for developing hydrophilic ligands, contributing to the selective separation of f‐block elements and is also beneficial for the formulation of stable, water‐soluble f‐block complexes.

## 2 Experimental Section

### 2.1 Materials and Characterizations

All chemicals and ultradry solvents, unless otherwise specified, were procured from Energy Chemical Inc. and utilized without additional purification. Other reagents were of analytical or chromatographic grade (Bei Jing TongGuang Fine Chemicals Company). **TODGA** was acquired from Qingdao Beitwall Technology Co., Ltd. and utilized directly without further purification. Milli‐Q water (18.2 MΩ·cm) was employed for all experimental procedures. Eu(NO_3_)_3_∙6H_2_O, La(NO_3_)_3_∙6H_2_O, and Lu(NO_3_)_3_∙6H_2_O (99.99% purity) for titration and single‐crystal experiments were obtained from Aladdin. Radioactive tracer stock solutions, ^241^Am, ^244^Cm, and ^152,145^Eu were provided by the Institute of Nuclear and New Energy Technology (INET). ***Caution*** *: ^241^Am, ^244^Cm and ^152,154^Eu* are highly radioactive and radiotoxic isotopes; improper handling may lead to severe health consequences. All experiments involving these isotopes were conducted in a dedicated radiological facility specifically designed for transuranic element studies.

The activity concentrations of radionuclides in the solution were determined using a Liquid Scintillation Spectrometer (Quantulus 1220, PerkinElmer) for precise radioactivity measurements. UV–vis absorption spectra were recorded on a Cary 6000i spectrophotometer (Agilent Inc.) at 298 K. Photoluminescence (PL) and photoluminescence excitation (PLE) spectra were recorded on a Hitachi F‐4500 spectrometer with 300 nm excitation wavelength and 5 nm emission slits for both excitation and emission. Nuclear Magnetic Resonance spectroscopy (NMR) was collected on a Varian‐600 spectrometer in deuterated dimethylsulfoxide (DMSO‐*d*6) with tetramethylsilane (TMS, 0.03% v/v) as the internal standard, unless otherwise specified. For NMR titrations, a 0.75 M DNO_3_/D_2_O solution was used. Ligand solubilities were quantified via both visual inspection and UV–vis absorption after 24‐h vortexing (200 rpm, 25 °C) of certain amount of ligand in 1 mL solvent, with “S” (soluble) defined as complete dissolution and “PS” (partially soluble) indicating incomplete dissolution. “PS” state was confirmed by direct observation while for “S” state, absorption spectra validations were conducted according to Beer's Law. Fourier transform infrared (FT‐IR) spectra of the ligands and complexes were performed and recorded on a Brucker Tensor 27 spectrometer. High resolution mass spectrometric (HRMS) analyses of the ligands and complexes were performed on a 12 T Solarix MALDI‐FT‐ICR MS (Bruker Daltonics). Single‐crystal X‐ray diffraction studies were conducted on a Rigaku Synergy‐R diffractometer with Cu Kα radiation (1.5406 Å) or Mo Kα radiation (0.771073 Å). And the empirical absorption correction was applied via φ‐scan. Cell parameters were determined by global refinement of all observed reflections. The crystal structure was solved by direct methods and refined by a full matrix least‐squares technique based on F^2^ using SHELXL‐97 program. All hydrogen atoms were refined isotropically, while non‐hydrogen atoms were refined anisotropically.

### 2.2 Solvent Extractions

Typical extraction experiments were conducted as follows: The aqueous phase was prepared by dissolving a specified amount of water‐soluble ligands (for experiments involving **Phen‐2DIC2OMe** and **Phen‐2DICy**) in nitric acid solutions of varying acidity, spiked with trace amounts of ^241^Am, ^244^Cm, and ^152,154^Eu. For **Phen‐2DIC2OMe** and **Phen‐2DICy** systems, the organic phase was prepared by dissolving a measured quantity of **TODGA** in *n*‐dodecane, whereas for **Phen‐2DIC2SMe**, the organic phase was **Phen‐2DIC2SMe** in *n*‐octanol. Subsequently, Equal volumes (0.5 mL) of the aqueous and organic phases were combined in sealed glass tubes and vigorously mixed for 30 min at 25 ± 1 °C using a vortex shaker in a temperature‐controlled water bath. After reaching extraction equilibrium, the two phases were separated by centrifugation at 3000 r min^−1^ for 2 min. Aliquots from both phases were sampled before and after extraction, and the concentrations of ^241^Am, ^244^Cm, and ^152,154^Eu were quantified using a Liquid Scintillation Spectrometer (Quantulus 1220, PerkinElmer). The distribution ratio (*D*) was defined as the ratio of radioactivity counts per unit volume in the organic phase to that in the aqueous phase. Separation factors (*SF*) were calculated as the ratio of *D* values for respective radionuclide pairs.

### 2.3 UV–vis Absorption Spectroscopy Titrations

The coordination behaviors of ligands with Eu(NO_3_)_3_ in nitric acid solutions was probed by UV–vis absorption spectroscopy titrations. All experiments were conducted under thermostatic conditions (25 ± 1 °C) to minimize temperature‐induced perturbations. A Cary 6000i UV–vis–NIR spectrophotometer (Agilent Inc.) equipped with a 1 cm quartz cuvette was used to record the absorption spectra during titrations. To maintain constant ionic strength, 0.1 m NaNO_3_ was added to the solutions. For titrations of **Phen‐2DIC2OMe** and **Phen‐2DICy**, a solution of 4.0 mM Eu(NO_3_)_3_ in 0.01 m HNO_3_ was incrementally titrated into 1.6 mL of 0.01 mm ligand in 0.01 m HNO_3_, and the absorption spectra of Eu(III) were monitored over the wavelength range of 250–370 nm. For **Phen‐2DIC2SMe**, a solution of 4.0 mm Eu(NO_3_)_3_ in methanol was titrated into 1.6 mL of 0.01 mm ligand in methanol, and the absorption spectra of Eu(III) were similarly monitored. After each addition, the mixture was vigorously agitated for 5 min at 25 ± 1 °C to ensure complete complexation before spectral recording. Preliminary kinetic studies confirmed that equilibrium was achieved under these conditions. The spectral data obtained from titrations were fitted using the nonlinear regression program *HypSpec* to derive speciation profiles and corresponding thermodynamic stability constants.

### 2.4 Time‐Resolved Laser Fluorescence Spectroscopy (TRLFS) Titrations

Photoluminescence (PL) and lifetime titration experiments of Eu(III) were conducted under thermostatic conditions (25 ± 1 °C) on an Edinburgh FLS‐1000 spectrophotometer equipped with a 450 W ozone‐free xenon arc lamp. A pulsed microsecond xenon lamp (150 W, pulse width ≈1 µs) served as the light source. To maintain constant ionic strength, 1.0 M NaNO_3_ was added to the solutions. For titrations of **Phen‐2DIC2OMe** and **Phen‐2DICy**, a solution of 10.0 mM ligand in 0.75 M HNO_3_ was incrementally titrated into 1.6 mL of 1.0 mM Eu(NO_3_)_3_ in 0.75 M HNO_3_. Eu(III) emission spectra were monitored between 550–750 nm (0.5 nm per step, 3 nm bandwidth) upon excitation at 394 nm (electronic level of ^5^L_6_, 2 nm bandwidth). After each addition, the mixture was agitated for 5 min at 25 ± 1 °C to ensure complete complexation, followed by recording of the PL spectra and the emission lifetime at 613 nm (corresponding to the ^5^D_0_→^7^F_2_ transition of Eu(III)). Decay data were analyzed using the software package integrated into the Edinburgh FLS‐1000 spectrophotometer, with goodness of fit assessed by minimizing the χ^2^ function and visually inspecting the weighted residuals.

### 2.5 Nuclear Magnetic Resonance Spectroscopy (NMR) Titrations

NMR titrations were conducted on a Varian‐600 spectrometer. For **Phen‐2DIC2OMe** and **Phen‐2DICy**, titrations were performed in 0.75 M DNO_3_/D_2_O. Aliquots of 100 mM La(NO_3_)_3_ and Lu(NO_3_)_3_ in 0.75 M DNO_3_/D_2_O were incrementally titrated into 0.5 mL of 10 mm ligand in 0.75 m DNO_3_/D_2_O. For **Phen‐2DIC2SMe**, Methanol‐*d*4 was used as the solvent, and aliquots of 100 mm La(NO_3_)_3_ and Lu(NO_3_)_3_ in Methanol‐*d*4 were titrated into 0.5 mL of 10 mM ligand in Methanol‐*d*4. After each addition, the NMR tube was inverted for 5 min to ensure complete mixing and complexation of the ligand with La(III) and Lu(III), achieving equilibrium. ^1^H NMR spectra were recorded after each titration step. Post‐titration, the stability of the ligand‐metal complexes under acidic conditions was continuously monitored.

### 2.6 IR Sample Preparations

Fourier transform infrared (FT‐IR) spectroscopy was conducted using a Brucker Tensor 27 spectrometer, covering a wavenumber range of 500–4000 cm^−1^ with a resolution of 4 cm^−1^. A methanol solution containing the ligand and Eu(NO_3_)_3_ at a 1:1 metal‐to‐ligand molar ratio was stirred at room temperature for 12 h and dried to yield a light‐yellow solid. For characterization, the sample was mixed with KBr (2 wt%) and pressed into pellets prior to analysis.

### 2.7 ESI‐MS Characterizations

High‐resolution mass spectrometry (HRMS) analysis of the ligand‐metal complex was conducted on a 12 T Solarix MALDI‐FT‐ICR MS (Bruker Daltonics). Prior to analysis, the ligand and metal were dissolved in methanol at a 1:1 molar ratio and stirred at room temperature for at least 12 h to ensure complete mixing. Samples were introduced via a 15 mL stainless steel syringe. Instrument parameters included a nebulizer gas pressure of 8 psi, a nitrogen flow rate of 5 L min^−1^, an ion source temperature of 200 °C, and a capillary voltage of 3500 V. Spectra were acquired in positive ion mode with broadband detection, typically covering a mass range of 50–1200. Peak *m/z* values and absolute intensities were processed using Bruker data analysis software.

### 2.8 Single Crystal X‐Ray Diffractions (SC‐XRD)

Single crystals of three kinds of ligand‐Eu(III) complexes were obtained via slow evaporation from a 1:1 (v/v) methanol‐isopropanol solution. To ensure complexation, 0.02 mmol of the ligand was dissolved in 2 mL of methanol, followed by the addition of 0.02 mmol of Eu(NO_3_)_3_·6H_2_O in 1 mL of methanol. The mixture was stirred at room temperature for 5 h, followed by evaporation to dryness, yielding a red‐fluorescent solid that confirmed the formation of the metal‐ligand complex. The resulting complex was redissolved in 2 mL of methanol, and 2 mL of isopropanol was carefully added. Platelet‐shaped crystals formed at the bottom of the vessel after two days of slow evaporation. Single‐crystal X‐ray diffraction data were collected on a Rigaku Synergy‐R diffractometer, with experimental details provided in the Materials and Characterization section.

### 2.9 DFT Calculations

The energy barriers for the rotations of carbonyl groups in **Phen‐2DIC2OMe** and **Phen‐2DICy** were illustrated by DFT calculations. Theoretical investigations were carried out with the hybrid density functional B3LYP and 6–31G (d, p) basis set in the Gaussian 09 program. The D3 (BJ) dispersion correction was applied to properly treat the weak interactions.^[^ ^13^ ^]^ After geometric optimization of the two ligands (from single crystal data), relaxed scan of the potential energy surface was performed by conducting the carbonyl group rotation side by side.

For the interpretation of the water solubilities for **Phen‐2DIC2OMe** and **Phen‐2DIC2SMe**. The same method was used for treating the weak interactions. The solvation effect was investigated through the polarizable continuum model in the Gaussian 09 program. The lattice energy was obtained by the difference between the energies of the crystal and single molecule. Both the atomic structures and lattice parameters were optimized with the CRYSTAL17 program.^[^ ^14^ ^]^ The differences in the water solubilities of the two ligands were quantitively compared by considering both the lattice energy and the solvation energies.

## 3 Results and Discussion

### 3.1 Ligand Design, Synthesis, and Solubility Demonstrations

Totally three ligands were synthesized and reported in the current work. Phenanthroline diimine framework was selected for its energy‐favored coordination architecture and its hydrogen bond formation ability at the imine sites.^[^ ^7^, ^12^, ^15^ ^]^ The 2‐methoxyethylamine (**Phen‐2DIC2OMe**) and 1,2‐bis(2‐aminoethoxy)ethane (**Phen‐2DICy**) substituted ligands were designed to investigate how ligand topology influences overall water solubility and extraction performance. At the same time, 2‐(methylthio)ethylamine group (**Phen‐2DIC2SMe**) was initially introduced to fine‐tune the second coordination sphere of the final complex. This modification would enhance the ligand's lipophilicity (as sulfur's less electronegativity and poorer hydrogen‐bond formation ability with respect to oxygen) while minimizing the impact on water solubility (sulfur and oxygen are in the adjacent position in periodic table). The goal was to exclude water and/or proton coordination, thereby improving acid resistance. Both **Phen‐2DIC2OMe** and **Phen‐2DIC2SMe** were readily prepared from *N*‐hydroxysuccinimide activated phenanthroline dicarboxylate (Scheme S3, Supporting Information) as crystalline powders with high yields. The cyclic counterparts of **Phen‐2DICy** was synthesized according to a modified procedure from that reported by Cruz.^[^ ^16^ ^]^ The chemical identities of all the ligands were solidly confirmed by ^1^H, ^13^C, 2D‐COSY NMR, HRMS spectroscopy (Figures S1–S9; Tables S1 and S2, Supporting Information) and single crystal X‐ray diffraction (SCXRD) (Tables S3–S5, Supporting Information). Close comparison of ^1^H NMR spectroscopy of **Phen‐2DIC2SMe** and **Phen‐2DICy** with respect to that of **Phen‐2DIC2OMe** indicated the deshielding effects of imine proton for the former two (Figures S10 and S11, Supporting Information). On the other side, the ^13^C NMR showed that no obvious shifts for the imine carbons (Figure S12, Supporting Information). Further single crystal analyses revealed that water mediate and intermolecular hydrogen bond network existed for **Phen‐2DICy** (Figure S13, Supporting Information) and **Phen‐2DIC2SMe** (Figure S14, Supporting Information) but no detectable hydrogen bonding in the crystal of **Phen‐2DIC2OMe** (Figure S15, Supporting Information). The crystal data explained the NMR shifts well but the reason for the different hydrogen bond networks were unclear at this stage. Furthermore, the introduction of sulfur atoms shielded both the adjacent hydrogens and carbons (Figures S11 and S12, Supporting Information) which could arise from the larger atomic size and the more diffused electrons for sulfur in comparison with oxygen. This could impact the metal binding affinity of the ligands as indicated in the HRMS of **Phen‐2DIC2OMe** and **Phen‐2DIC2SMe** (Figures S4 and S8; Tables S1 and S2, Supporting Information): the larger and more electron diffused sulfur containing ligands preferred the formation of dimeric architectures with mono charged cations (H^+^, Na^+,^ and K^+^).

As for the ligand photophysics, both the topological (**Phen‐2DICy**) and the heteroatomic (**Phen‐2DIC2SMe**) modifications seemed to have little effect on the absorption, emission, and energy levels of the final ligands (Figures S16–S18, Supporting Information), indicating the predominant role of phenanthroline diimine framework in the photophysical properties. On the contrary, solubility tests revealed that ligand architectures had great impacts on the overall water solubilities (Figure S19, Supporting Information). A clear correlation emerges between HNO_3_ concentration and ligand solubility. **Phen‐2DIC2OMe** displayed over 200 mM solubility in 1–3 M HNO_3_ and an approximate of 30 mM in environmental benign solvent of octanol.^[^ ^12c,e^ ^]^ Cyclizing at the far end of the binding pocket, **Phen‐2DICy** displayed compromised solubilities in both 1 M HNO_3_ (ca. 20 mM) and octanol (ca. 10 mM). This threshold behavior suggests that **Phen‐2DICy** solubility depends critically on acid concentration. SCXRD data revealed the decreased solubilities were not arose from the π‐stacking of the phenanthroline skeleton (Figures S20–S21, Supporting Information). The introduction of sulfur atom (**Phen‐2DIC2SMe**) resulted in the totally loss of water (acid) solubility and a similar octanol solubility as that of **Phen‐2DICy**. In the following discussions, we will focus on the extraction performances of these chemical modifications and then discuss from molecular bias on how the ligand structural alternation could impact the metal coordination/extraction behaviors.

### 3.2 Rotation Barriers Impact Ligand Masking Efficiencies in Aqueous Phase

**Figure 1:**
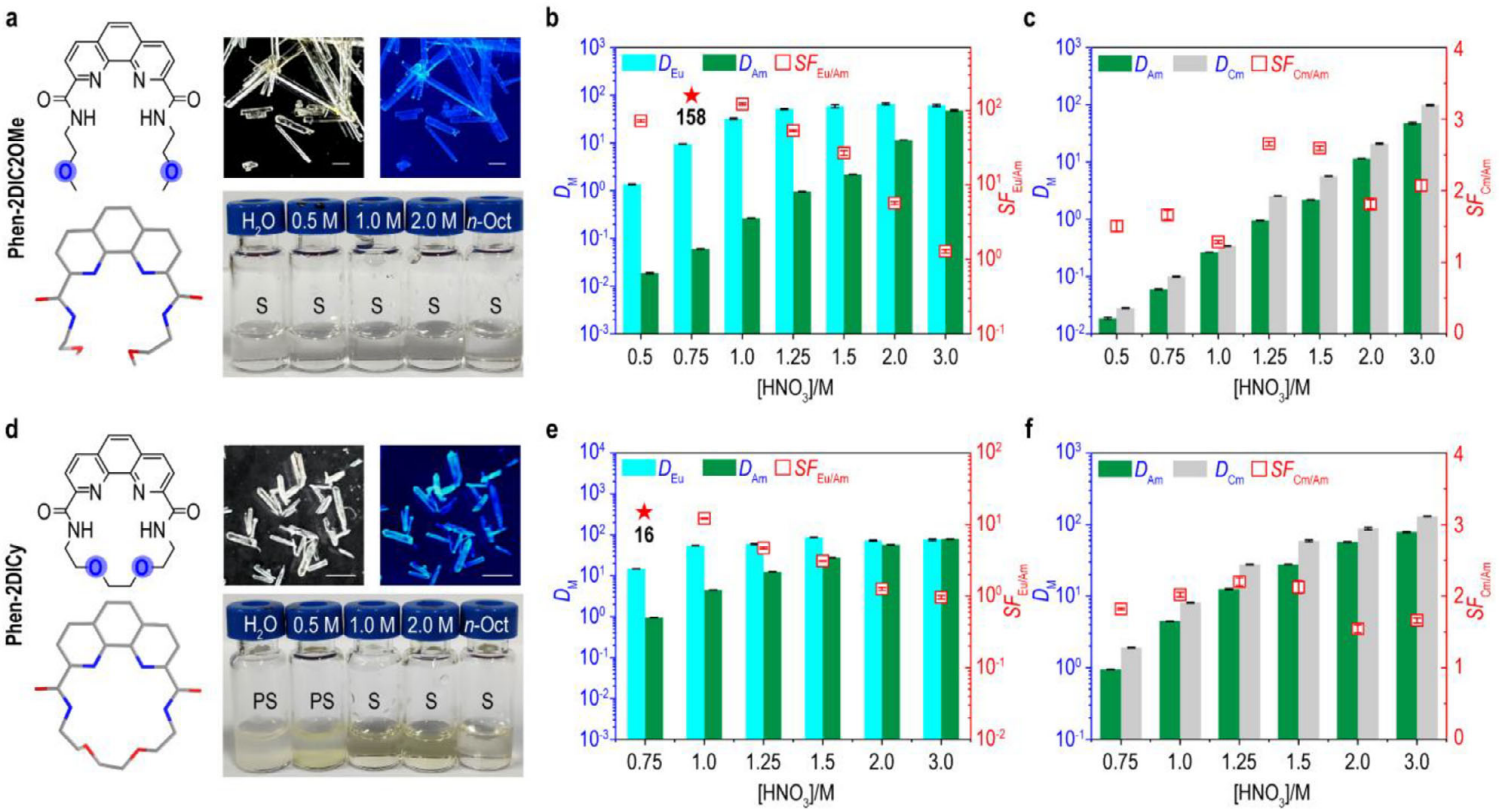
The effects of ligand topology on the ligand solubility and extraction performances. a) Chemical and single crystal structures, images under white light and 365 nm excitation and solubility tests in different concentration of HNO_3_ and octanol for **Phen‐2DIC2OMe**. Abbreviations of S and PS represented soluble and partially soluble respectively. All ligand concentration was 10 mM. Distribution ratios (*D*_M_) and separation factors (*SF*) obtained in the extraction of Eu(III)/Am(III) (b) and Cm(III)/Am(III) (c) by **TODGA** (in the organic phase) and with **Phen‐2DIC2OMe** in the aqueous phase as functions of HNO_3_ concentrations. Experiment conditions: Organic phase (O): 100 mm **TODGA** in dodecane. Aqueous phase (A): 10 mm **Phen‐2DIC2OMe** in HNO_3_. O/A = 1; Vortex shaker (50 Hz) for 30 min at 25 °C. d–f) The same but for **Phen‐2DICy**. Scale bars in all images were 10 mm. Error bars were averaged from three duplicated experiments.

As noted in the previous section, **Phen‐2DIC2OMe** and **Phen‐2DICy** were designed to elucidate the ligand topology on water solubility and masking performances. The two ligands were chemically identical and topological different. The cyclization at the far end of the tetradentate binding pocket resulted in a six‐coordination geometry similar to that of 18‐crown‐6 despite incorporation of a more rigid phenanthroline framework. Both **Phen‐2DIC2OMe** and **Phen‐2DICy** formed rectangle crystals from direct evaporation of their methanol solutions (**Figure** **1**a,d). Compared to the hydroxyl‐group counterpart of **Phen‐2DIC2OMe**, the introduction of two methyl group did not impact the water solubility too much (20 mM for **Phen‐2DIC2OH**).^[^ ^12f^ ^]^ **Phen‐2DIC2OMe** was readily soluble in water with a concentration up to 10 mm (Figure 1a). Liquid‐liquid extraction experiments with **Phen‐2DIC2OMe** as aqueous masking agent and **TODGA**/*n*‐dodecane as the organic phase indicated that **Phen‐2DIC2OMe** was effectively selective for Am(III)/Eu(III) in HNO_3_ from 0.5 M to 2.0 M with maximum separation factor (*SF*_Eu/Am_) of 158 (0.75 M HNO_3_, *D*_Am_ = 0.06, *D*_Eu_ = 9.5), which was one of the highest reported value under high‐acidity conditions (of over 0.5 M, Table S6, Supporting Information).^[^ ^2^, ^7^, ^9^, ^10^, ^11^, ^12^, ^17^ ^]^ The slightly decreased *SF*_Cm/Am_ (also the shielding effects as reflected from the distributions for both Am and Cm) indicated the important role of hydroxyl groups which might require further single crystal data support. While when the two methyl groups were coupled together, the cyclic ligand of **Phen‐2DICy** could only dissolve in acid with concentration of higher than 0.75 m HNO_3_ (Figure 1d). At the meantime, **Phen‐2DICy** displayed a tenfold decreased Am(III) masking ability with respect to **Phen‐2DIC2OMe** which lead to an overall diminished *SF*_Am/Eu_ of 16 (Figure 1e). Similar decreasing for Cm(III) binding ability was observed as that for Am(III) thus indicated the cyclization have great impacts on the actinide masking ability of the hydrophilic ligands. Efforts from adding NaNO_3_ (Figures S22 and S23, Supporting Information) and increasing the ligand concentrations (Figures S24 and S25, Supporting Information) were all failed to recover the separation performances of **Phen‐2DICy**. Stoichiometric analysis via linear fits of distribution ratios versus ligand concentrations indicated that predominant formation of 1/1 and mixture of 1/1 and 1/2 species for both **Phen‐2DIC2OMe** and **Phen‐2DICy** with Eu(III) and Am(III) respectively (Figures S26 and S27, Supporting Information). Further extraction kinetics showed that both **Phen‐2DIC2OMe** and **Phen‐2DICy** displayed rapid coordination equilibrium within 5 min (Figures S28 and S29, Supporting Information).

**Figure 2:**
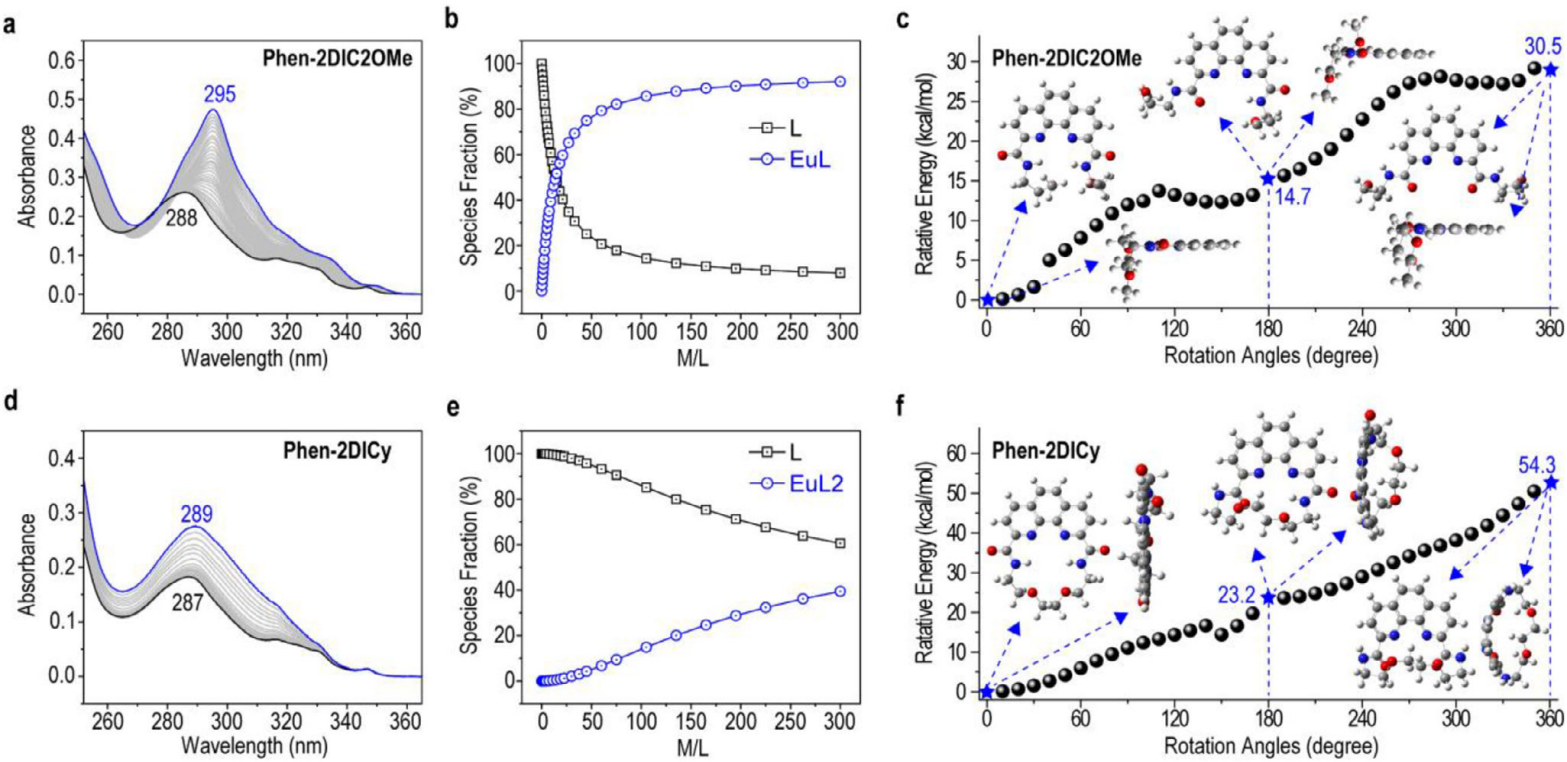
Solution coordination behaviors and rotation barriers analyses for **Phen‐2DIC2OMe** (a‐c) and **Phen‐2DICy** (d‐f). Absorption spectra titrations of **Phen‐2DIC2OMe** (a) and **Phen‐2DICy** (d) with Eu(NO_3_)_3_ in aqueous HNO_3_ (0.01 m) solution with the ion strengths controlled by 0.1 m NaNO_3_. Ligand and metal concentrations were 0.01 and 4 mm respectively. Species distributions (b, e) derived from panel (a, d). DFT calculations showing the relative energy changes for **Phen‐2DIC2OMe** (c) and **Phen‐2DICy** (f) when both the carbonyl groups rotated in order to accommodate the metal cations. The optimized ligand structures from both top‐view and side‐view were also given at the initial state 0° (free ligand), 180° (half coordinated), and 360° (fully coordinated) with the energies given in blue.

To elucidate the pronounced differences in extraction performance between **Phen‐2DIC2OMe** and **Phen‐2DICy**, multiple spectroscopic titrations were employed to investigate the solution coordination behaviors. As depicted in **Figure** **2**, UV–vis absorption titrations were utilized to investigate the distinct coordination behaviors of **Phen‐2DIC2OMe** and **Phen‐2DICy** with Eu(NO_3_)_3_ in 0.01 M HNO_3_ with ionic strength controlled by 0.1 M NaNO_3_. Upon adding Eu(III) into the **Phen‐2DIC2OMe** solution, the low‐energy absorption peak at 288 nm gradually shifted to 295 nm with increasing absorption intensity (Figure 2a). This was typical sign of strong coordination and arose from metal‐ligand charge transfer processes.^[^ ^3^, ^5^ ^]^ Fitting the titration curves with *HypSpec* program giving the species evolution under the titration conditions. Predominant 1/1 species were identified which was consistent with that of stoichiometric analysis. On the other side, the absorption spectra of **Phen‐2DICy** displayed weak metal‐ligand interaction with only 2 nm of peak shifts (Figure 2d). The best‐fitted results did not self‐converge even with the addition of 300 equivalent of Eu(III) with the predominant species determined to be metal/ligand ratio of 1/2 (Figure 2e). Furthermore, NMR titrations under the same conditions as that of extraction experiments confirmed the comparative weak interactions of **Phen‐2DICy** toward f‐block metal cations (Figures S30–S33, Supporting Information). To reveal how ligand topologies affect the overall metal binding, geometry optimizations were conducted for both **Phen‐2DIC2OMe** and **Phen‐2DICy**. As depicted in Figure 2c,f, energies changes with respect to the free ligand states were calculated while gradually rotation of the carbonyl groups to mimic the ligand geometry changes during metal coordination. Representative ligand geometries were given from both top and side views along with the corresponding free energies. From the DFT calculations, it's obvious that the cyclization increased the energy barriers for the coordination‐favored carbonyl‐switched geometry (1/3 enhanced barriers per each carbonyl for **Phen‐2DIC2OMe** with respect to **Phen‐2DICy**). Close ligand geometry analyses showed that the observed energy barriers arose from the deformation of the phenanthroline diimine framework (side views for **Phen‐2DICy** in Figure 2f). At this stage, we conclude that the rigid conformation of **Phen‐2DICy** increases the energy barriers associated with metal coordination, particularly for the larger actinide cations. This hinders the ligand's ability to effectively bind Am(III), ultimately leading to reduced separation factors. These results unequivocally showed that terminal group topology‐except core preorganization‐as the dominant factor governing metal selectivity. The 53.3% and 88.5% increase in rotational energy barriers for two carbonyl groups in **Phen‐2DICy** (Figure 2f) directly impedes metal ion binding efficiency. Which positions terminal groups as conformational “selectivity switches” that control metal access to the preorganized core.

**Figure 3:**
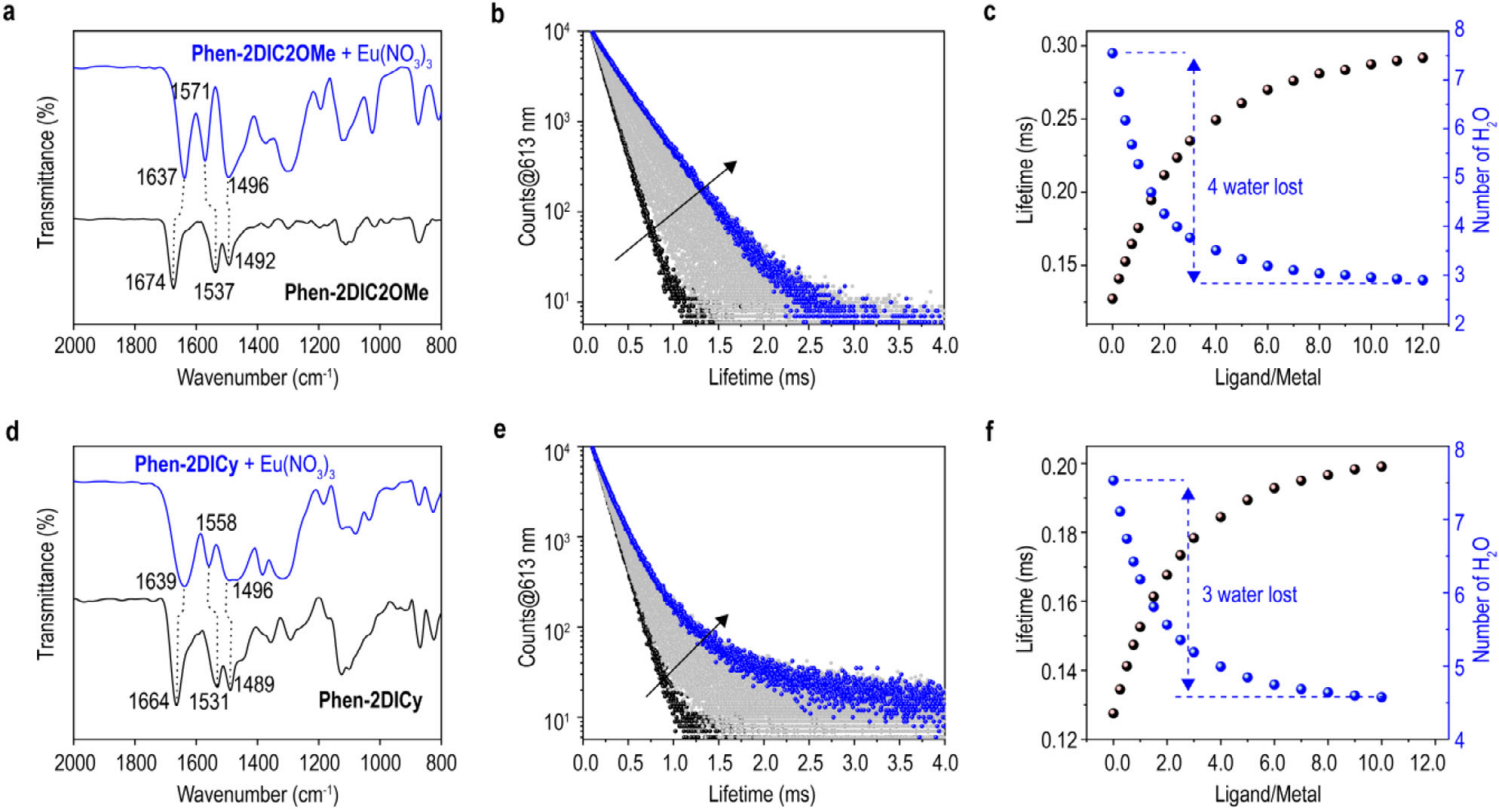
Coordination modes analyses for **Phen‐2DIC2OMe** and **Phen‐2DICy**. Zoomed‐in IR spectra for **Phen‐2DIC2OMe** (a) and **Phen‐2DICy** (d) before and after Eu(III) coordination. Lifetime decay curves (b, e) and calculated water molecules in the inner coordination sphere of Eu(III) (c, f) monitored at the most intense emission peak of 613 nm from ^5^D_0_ to ^7^F_J_ (J = 2) Eu(III)‐centered transition. Experiment condition: C_Ligand_/C_Eu(III)_ = 10 mM/1 mM in 0.75 M HNO_3_ with 1.0 M NaNO_3_. Totally 2.0 mL of titrant was added to V_0_ = 1.6 mL of initial solution. The numbers of the total water loss were given in panel c and f.

The coordination modes for **Phen‐2DIC2OMe** and **Phen‐2DICy** were further examined by infrared spectroscopy (IR) and time‐resolved laser fluorescence spectroscopy (TRLFS) titrations. As shown in **Figure** **3**a,d (full spectra given in Figures S34 and S35, Supporting Information), upon coordination with Eu(III), the carbonyl groups on both **Phen‐2DIC2OMe** and **Phen‐2DICy** shifted toward lower energy regions because of the electron donating natures from C═O double bonds to the empty metal f orbitals.^[^ ^5^, ^7^, ^12^, ^18^ ^]^ The magnitudes of the shifting could be used as standards for the coordination strengths with 37 cm^−1^ for **Phen‐2DIC2OMe** in comparison to 25 cm^−1^ for **Phen‐2DICy** which was consistent with the absorption titration results reflecting the weaker coordination of **Phen‐2DICy**. At the same time, the IR signals were much broader for the carbonyl groups in **Phen‐2DICy** after coordination with Eu(III) (FWHM of 23 and 3 cm^−1^ for respectively for the differences of Gaussian fit at carbonyl groups before and after coordination for **Phen‐2DICy** and **Phen‐2DIC2OMe**, Figure S36, Supporting Information) which could origin from the existence of multiple chemical environments of carbonyl groups in **Phen‐2DICy** than that in **Phen‐2DIOMe**. Furthermore, the estimated water molecules from the TRLFS titrations (Figure 3b,c,e,f; Note S1; Figure S37, Supporting Information) showed totally 4 and 3 waters were lost during the coordination processes of **Phen‐2DIC2OMe** and **Phen‐2DICy** with Eu(III) cations. From all these results, we speculated that for **Phen‐2DIC2OMe**, predominant 1/1 ligand/metal species formed during extraction with Eu(III) and **Phen‐2DIC2OMe** acted as a tetradentate ligand (*ONNO* biting). While in the case of **Phen‐2DICy**, the topological strains restricted the rotation of carbonyl groups resulting in a bidentate‐like ligand for **Phen‐2DICy**. In order to fulfil the coordination sphere of Eu(III), the other **Phen‐2DICy** might behave as a monodentate ligand coordinating to the metal center probably through one of the two carbonyl groups which gave the different carbonyl signals in IR spectra (overall L/M of 2 as that observed in absorption titrations).

### 3.3 Non‐Coordinating Sulfur Atoms alter the Ligand Water Solubilities

**Figure 4:**
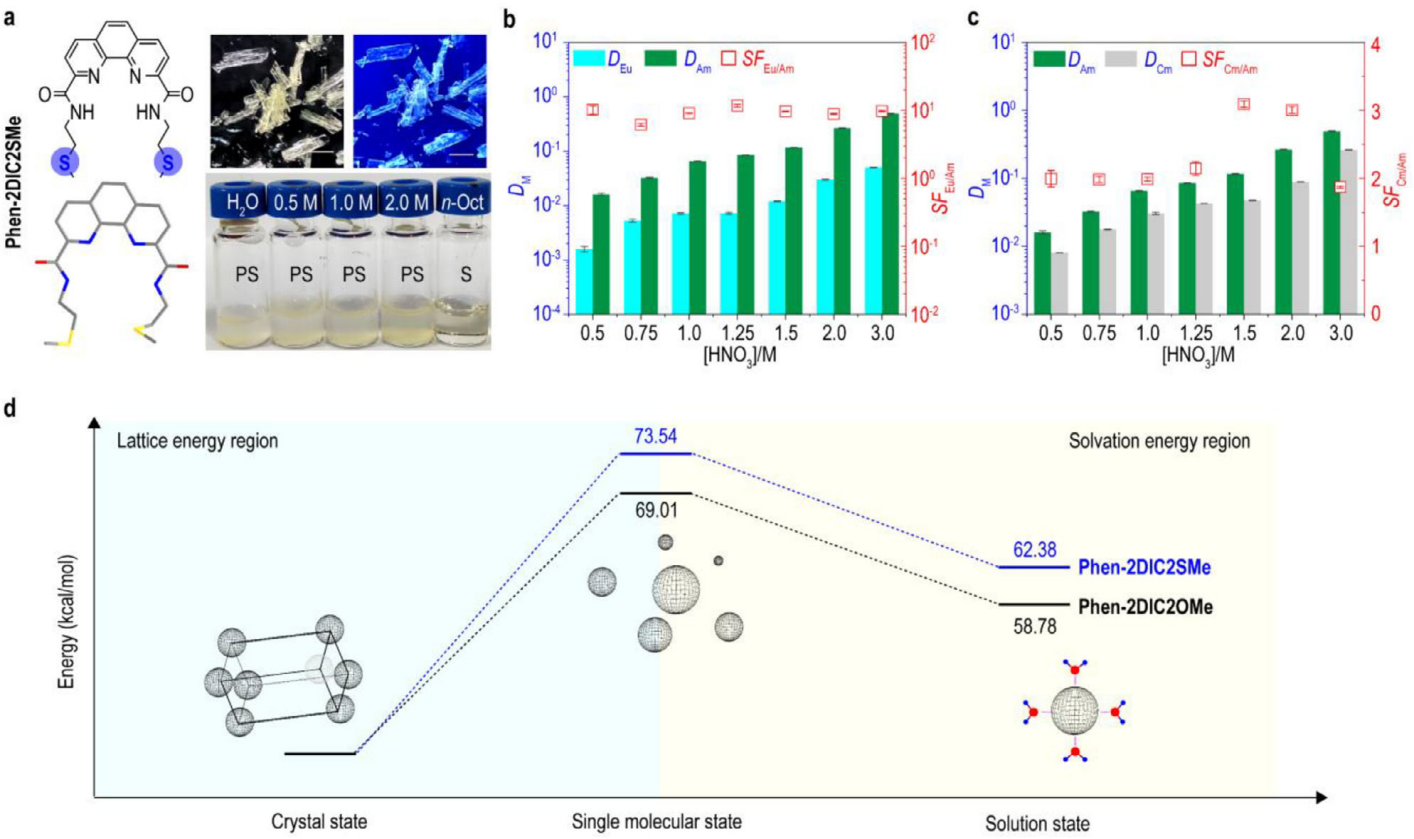
The effects of non‐coordinating sulfur atoms on the ligand solubility and extraction performances. a) Chemical and single crystal structures, images under white light and 365 nm excitation and solubility tests in different concentration of HNO_3_ and octanol for **Phen‐2DIC2SMe**. Scale bars in the all the images were 10 mm. Distribution ratios (*D*_M_) and separation factors (*SF*) obtained in the extraction of Eu(III)/Am(III) (b) and Cm(III)/Am(III) (c) by **Phen‐2DIC2SMe** as functions of HNO_3_ concentrations. Experiment conditions: Organic phase (O): 10 mm **Phen‐2DIC2SMe** in octanol. Aqueous phase (A): different concentrations of HNO_3_. O/A = 1; Vortex shaker (50 Hz) for 30 min at 25 °C. (d) DFT calculations showing the lattice energy and solvation energy for both **Phen‐2DIC2OMe** and **Phen‐2DIC2SMe**. Scale bars in all images were 10 mm. Error bars were averaged from three duplicated experiments.

Aware of the rotation barriers introduced by the topological strained in **Phen‐2DICy**, we turned to heteroatom substitution to fine‐tune the ligand extraction performances. Recall the basis for lanthanide/actinide separation: the prevailing consensus is that the larger atomic and ionic radii of actinides enable greater bond covalency when interacting with ligands.^[^ ^19^ ^]^ In this context, sulfur‐containing chelators show great potential for facilitating the separation process.^[^ ^10^, ^20^ ^]^ The most successful example was the bis(2,4,4‐trimethylpentyl)dithiophosphinic acid (**Cyanex 301**) which gave a *SF*_Am/Eu_ of over 5000.^[^ ^21^ ^]^ In fact, sulfur‐containing amino acids, such as cysteine and methionine, are widely recognized as essential building blocks in biological systems due to their nucleophilicity, redox activity, and metal‐binding capabilities.^[^ ^22^ ^]^ Additionally, the long‐range, weak intermolecular interactions mediated by sulfur atoms have been reported to play a critical role in modulating the second coordination sphere of proteins, particularly in metalloenzymes.^[^ ^22c,d^ ^]^ Thus, the 2‐(methylthio)ethylamine analogue of **Phen‐2DIC2SMe** was prepared. Substitution of methylthio‐ group preserved the crystallinity of **Phen‐2DI** core, **Phen‐2DIC2SMe** was afforded as rectangular single crystals when precipitated from the reaction mixture (**Figure** **4**a). SCXRD results showed the introduction of sulfur atom at the far end of the ligand switched the molecular packing in the crystal cells (Tables S3 and S5, Supporting Information). Both **Phen‐2DIC2OMe** and **Phen‐2DIC2SMe** belonged to orthorhombic space group with higher symmetry subgroups of Pbca (glide planes and center of symmetry) for **Phen‐2DIC2SMe** with respect of P2_1_2_1_2_1_ (three 2_1_ screw axes) for **Phen‐2DIC2OMe**. The tighter and more symmetrical packing of **Phen‐2DIC2SMe** could be the molecular origin for the observed solubility as discussed in the previous section. As **Phen‐2DIC2SMe** was negligible insoluble in aqueous (acid) solution (< 0.1 mM), the Lns(III)/Ans(III) and Ans(III)/ Ans(III) separation performances were illustrated in octanol. Extraction experiments revealed relatively mediocre extraction and separation capabilities of **Phen‐2DIC2SMe** with maximum separation factors (*SF*) of ≈12 for Eu(III)/Am(III) (*D*_Am_ = 0.085, *D*_Eu_ = 0.007, Figure 4b) and ≈3 for Cm(III)/Am(III) (*D*_Am_ = 0.12, *D*_Cm_ = 0.04) in 0.5‐3.0 M HNO_3_ (Figure 4c). We noticed that the sulfur substitution at the noncoordinating sites not only diminished the ligand water solubility, at the same time, the extraction ability was crippled. Comparison of **Phen‐2DIC2SMe** with our previous reports of **DIPhen‐C4** (similar ligand structure while S was changed to C for **DIPhen‐C4**), about tenfold decreased metal distributions were observed for both Eu(III) and Am(III) at 3 m HNO_3_ with the same ligand concentration of 10 mm.^[^ ^12c^ ^]^ This results clearly showed that even sulfur atoms were not directly involved in the metal coordination, they somehow impact the metal affinity through weaken the metal‐oxygen bonds in the final complexes (Figure S38; Tables S7–S9, Supporting Information). As it's generally believed the metal‐oxygen bonds were important for the ligand affinity toward the metal cations,^[^ ^3^, ^5^, ^12^, ^23^ ^]^ the weakened metal‐oxygen bonds could be the reason for the low extraction distributions of **Phen‐2DIC2SMe**. This hypothesis was further confirmed by the ESP calculations on both ligands which clearly showed the reduced electron density of oxygen in **Phen‐2DIC2SMe** (Figure S39, Supporting Information).

To better understand how the noncoordinating atom (S) impact the water solubility of the ligand in the case of **Phen‐2DIC2SMe**, the whole energy landscapes of the ligand dissolution processes were considered. As depicted in Figure 4d, the dissolution process of a block crystalline ligand was divided into lattice energy region where compact stacking molecules in the crystal collapsed into free single molecules (gas phase); the second process was solvation energy region during which the free molecules in the gas phase interacted with the solvent molecules to be physically dissolve the ligand into the solvent system (water in our case). The optimized ligands structures for **Phen‐2DIC2OMe** and **Phen‐2DIC2SMe** were from SCXRD and the lattice parameters were optimized with the CRYSTAL17 program. The results clearly demonstrated the predominant role of the lattice energy for the overall dissolution of the ligands where ‐69.01 and ‐73.54 kcal mol^−1^ were required for **Phen‐2DIC2OMe** and **Phen‐2DIC2SMe** respectively. Even the energy‐favored solvation processes were observed for **Phen‐2DIC2SMe** with respect to **Phen‐2DIC2OMe** (11.16 and 10.23 kcal mol^−1^), the energy difference of 0.93 kcal mol^−1^ did not compensate the large lattice energy difference of 4.53 kcal mol^−1^. We proposed that the larger lattice energy for **Phen‐2DIC2SMe** could arise from the inter‐ligand hydrogen bonds between the imine and the carbonyl groups which were not observed in the case of **Phen‐2DIC2OMe** (Figures S40 and S20, Supporting Information). Collectively, these results demonstrated that non‐coordinating terminal groups‐even when spatially away from the binding core‐could exert decisive control over ligand performance. The methylthio (‐SMe) group in **Phen‐2DIC2SMe** simultaneously crippled water solubility (via enhanced lattice energy, Figure 4d) and metal affinity (through weakened M─O bonds, Figure S38, Supporting Information), demonstrating that terminal functionality operates as an independent design axis orthogonal to the preorganized coordination site. To the best of knowledge, our work represents the first detailed illustration of the relationship between molecular structures with the water solubility for hydrophilic f‐block element coordination.

## 4 Conclusion

In this study, we have examined a series of phenanthroline diimine (**Phen‐2DI**) ligands to uncover structure‐property relationships that influence both aqueous solubility and metal coordination selectivity. Systematic modifications revealed that topological changes, while preserving solubility, can significantly increase rotational barriers, thereby reducing binding affinity and selectivity for Am(III) over Eu(III) and Cm(III). Conversely, seemingly benign modifications‐such as the introduction of a non‐coordinating methylthio group‐unexpectedly compromised water solubility. These findings highlight the sensitivity of ligand performance to subtle structural changes especially for hydrophilic ligands. Through combined experimental (solution‐ and solid‐state titrations) and computational (DFT) approaches, we elucidated how steric and electronic features govern ligand preorganization, solvation, and f‐block metal affinity and the effect of noncoordinating atoms on ligand water solubility. This work demonstrates that achieving optimal ligand performance requires fine‐tuning both electronic environments and conformational flexibility. Specifically, based on this work, it is recommended to preferentially choose flexible and hydrogen‐bonding terminal groups rather than sulfur‐containing‐groups for hydrophilic ligand design in order to ensure good solubility in acidic media while maintaining the pre‐organized N and O donor core with low rotational barrier in order to balance selectivity and conformational adaptability. Ultimately, this study provides valuable design principles for the development of next‐generation hydrophilic ligands that can operate under acidic conditions and enable the efficient, selective separation of f‐block elements. These insights are expected to accelerate the development of more effective nuclear waste reprocessing technologies and facilitate the formulation of stable, water‐soluble complexes for bioimaging and radiopharmaceutical applications (as water solubility and complex stability are also the two critical criteria for such applications).

## Conflict of Interest

The authors declare no conflict of interest.

## Author Contributions

B.L., Y.K., Z.‐Y.Z., L.‐D.W., H.‐Y.L., and Y.‐X.G. performed data collection, investigation, and validation. G.W. performed technical support, calculation. X.‐Y.T., L.W., and C.X. performed supervision and data analysis. X.‐Y.T., L.W., and C.X. performed conceptualization, funding acquisition, and project administration. B.L. and L.W. wrote the original draft. Y.K., X.‐Y.T., L.W., and X.C. reviewed and edited. All authors have given their approval to the final version of the manuscript. B.L., Y.K., and Z.Z. contributed equally to this work.

## Supporting information



Supporting Information

Supporting Information

## Data Availability

The data that support the findings of this study are available from the corresponding author upon reasonable request.
