# Supplementary material for: On the Design of Effective Water‐Soluble Actinide‐Masking Ligands Through Ligand Structure Modulation
Source: Adv Sci (Weinh). 2025 Aug 21;12(42):e12292. doi: 10.1002/advs.202512292 (PMC12622539; doi:10.1002/advs.202512292)
Supplement: Supplementary file 1 — Supporting Information [file ADVS-12-e12292-s002.docx]

**Supporting information for:**

**On the Design of Effective Water-Soluble Actinide-Masking Ligands through Ligand Structure Modulation**

Bin Li,^a, b, ¶^ Yu Kang,^b, ¶^ Ziyi Zhang,^b, c, ¶^ Ludi Wang,^b^ Haoyu Li,^b^ Yuxiao Guo,^a^ Guo Wang,^b^ Li Wang^b, *^, Xiaoyan Tang,^c, *^ and Chao Xu^a, *^

^a^ Institute of Nuclear and New Energy Technology, Tsinghua University, Haidian District, Beijing, 100084, China;

^b^ Department of Chemistry, Capital Normal University, Haidian District, Beijing, 100048 China;

^c^ Beijing National Laboratory for Molecular Sciences, Key Laboratory of Polymer Chemistry and Physics of Ministry of Education, Centre for Soft Matter Science and Engineering, College of Chemistry and Molecular Engineering, Peking University, Haidian District, Beijing, 100871, China;

^¶^ B. Li, Y. Kang and Z. Zhang contributed equally to this work.

^*^ Corresponding authors: liwang862011@gmail.com (Li Wang); xiaoyan.tang@pku.edu.cn (Xiaoyan Tang); xuchao@tsinghua.edu.cn; (Chao Xu)

KEYWORDS: f-Block element; Water-soluble; Selective coordination; Ligand design; Liquid-liquid separation

Scheme S1. Chemical structures for the representative lipophilic Am(III)/Eu(III) separation agents mentioned in the main text.

Scheme S2. Chemical structures for the representative hydrophilic Am(III)/Eu(III) separation agents mentioned in the main text.

**Ligand Syntheses and Characterizations**

Scheme S3. Synthesis procedures for **Phen-2DIC2OMe**, **Phen-2DIC2SMe** and **Phen-2DICy**.

Syntheses of **Phen-2DIC2OMe** and **Phen-2DIC2SMe**

Bis(2,5-dioxopyrrolidin-1-yl)1,10-phenanthroline-2,9-dicarboxylate was prepared following the procedures in in the work we reported previously.[1]

To a suspension of bis(2,5-dioxopyrrolidin-1-yl)1,10-phenanthroline-2,9-dicarboxylate (4.62 g, 10 mmol) in 30 mL DMSO was added either 2-methoxyethylamine (2.25 g, 30 mmol, 3 eq.) or 2-(methylthio)ethylamine (2.74 g, 30 mmol, 3 eq.). Upon sonication, the solution turned into a clear orange-yellow solution. Subsequently, 0.2 mL triethylamine (Et_3_N) was added as catalyst, and the mixture was stirred at room temperature (298 K) for 24 hours.

For **Phen-2DIC2OMe**, after completion of the reaction, water was added into the reaction mixture, followed by multiple extractions with dichloromethane (CH_2_Cl_2_). The product was purified by column chromatography (eluent: CH_2_Cl_2_:CH_3_OH = 30:1) followed by rotary evaporation afforded the product as white crystals with yield of 85% (3.23 g). ^1^H NMR (600 MHz, Chloroform-*d*): δ 9.03 (s, 1H), 8.58 (d, *J* = 8.2 Hz, 1H), 8.43 (d, *J* = 8.2 Hz, 1H), 7.91 (s, 1H), 3.81 (m, 2H), 3.71 (t, *J* = 5.4 Hz, 2H), 3.44 (s, 3H).

^13^C NMR (151 MHz, Chloroform-*d*) δ 164.43, 149.71, 144.16, 137.65, 130.42, 127.69, 121.42, 71.18, 58.75, 39.33.

HRMS [C_20_H_23_N_4_O_4_^+^] ([L+H^+^]), calculated for 383.1714, found 383.1713. Detailed analyses were given in Table S1.

For **Phen-2DIC2SMe**, water was introduced to the reaction mixture upon completion, resulting in the precipitation of a light yellow layered crystalline solid. The product was collected after filtration, dried in air, analytical pure product with a yield of 72.4% (3.0 g) was readily afforded. ^1^H NMR (600 MHz, Chloroform-*d*): δ 9.07 (t, *J* = 6.0 Hz, 1H), 8.58 (d, *J* = 8.2 Hz, 1H), 8.43 (d, *J* = 8.2 Hz, 1H), 7.91 (s, 1H), 3.84 (m, 2H), 2.89 (t, *J* = 6.6 Hz, 2H), 2.24 (s, 3H).

^13^C NMR (151 MHz, Chloroform-*d*) δ 164.27, 149.62, 144.21, 137.76, 130.50, 127.75, 121.44, 37.98, 34.02, 15.22.

HRMS [C_20_H_23_N_4_O_2_S_2_^+^] ([M+H^+^]), calculated for 415.1257, found 415.1255. Detailed analyses were given in Table S2.

Syntheses of **Phen-2DICy**

**Phen-2DICy** was synthesized by a modified procedure as that reported by Cruz.[2] Briefly, **Phen-2COOMe** (300 mg, 2 mmol) was dissolved in 20 mL chloroform-ethanol (v/v = 1/1). To which 1,2-bis(2-aminoethoxy)ethane (4.4 g, 30 mmol) was added. The reaction mixture was left string at room temperature for 24 hours before the solvent was removed under vacuum. Methanol was added to the crude product to precipitate the pure product which was then washed with ether and dried in air. Pale yellow crystals were afforded with a yield of 40% (304 mg). ^1^H NMR (600 MHz, Chloroform-*d*) δ 8.99 (s, 1H), 8.62 (d, *J* = 8.2 Hz, 1H), 8.43 (d, *J* = 8.2 Hz, 1H), 7.92 (s, 1H), 3.88 (d, *J* = 4.1 Hz, 2H), 3.78 – 3.74 (m, 4H).

Figure S1 ^1^H NMR spectrum of **Phen-2DIC2OMe** in CDCl_3_*.*

Figure S2 ^13^C NMR spectrum of **Phen-2DIC2OMe** in CDCl_3_*.*

Figure S3 2D ^1^H-^1^H COSY spectrum of **Phen-2DIC2OMe** in CDCl_3_*.*


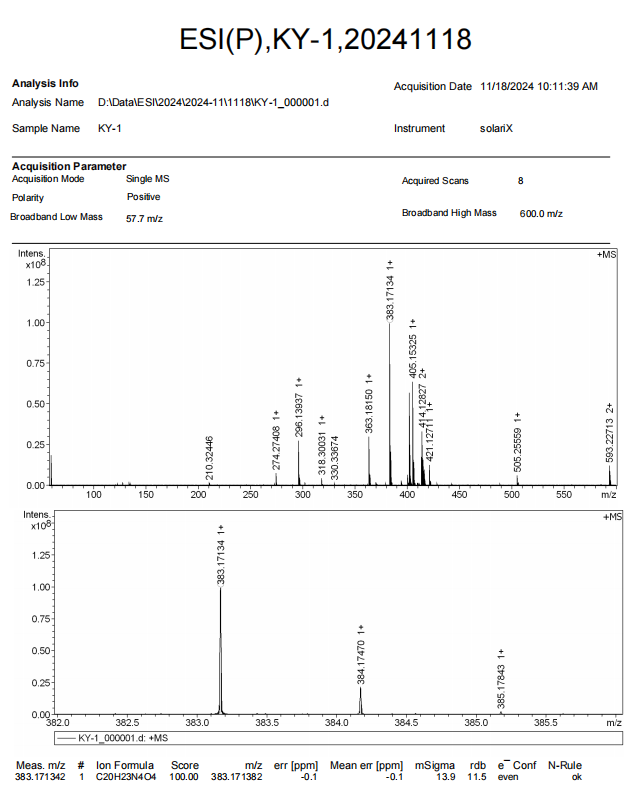


Figure S4 ESI-MS spectrum of of **Phen-2DIC2OMe***.*

Table S1 Analysis of ESI-MS for **Phen-2DIC2OMe**

| Speculated formula | Calculated MS | Measured MS |
| --- | --- | --- |
|   major peak | 383.1714 | 383.1713 |
|   major peak | 405.1533 | 405.1533 |
|  | 421.1273 | 421.1271 |

Figure S5 ^1^H NMR spectrum of **Phen-2DIC2SMe** in CDCl_3_*.*

Figure S6 ^13^C NMR spectrum of **Phen-2DIC2SMe** in CDCl_3_*.*

Figure S7 2D ^1^H-^1^H COSY spectrum of **Phen-2DIC2OMe** in CDCl_3_*.*


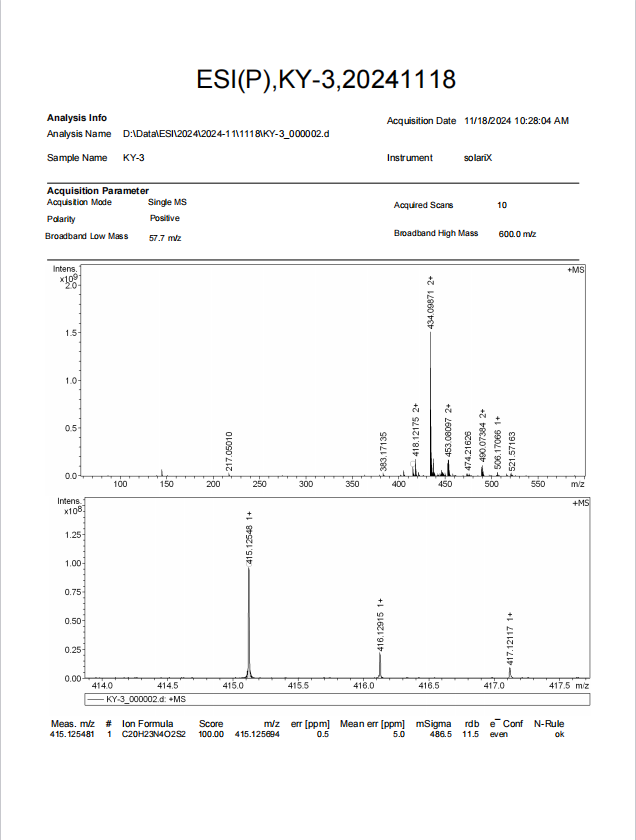


Figure S8 ESI-MS spectrum of of **Phen-2DIC2SMe***.*

Table S2 Analysis of ESI-MS for **Phen-2DIC2SMe**

| Speculated formula | Calculated MS | Measured MS |
| --- | --- | --- |
|  | 415.1257 | 415.1255 |
|   major peak | 434.1037 | 434.0987 |
|  | 453.0816 | 453.0810 |

| Table S3 Crystal data and structure refinement for **Phen-2DIC2OMe** | |
| --- | --- |
| Identification code | **Phen-2DIC2OMe** |
| CCDC | 2445307 |
| Empirical formula | C_20_H_22_N_4_O_4_ |
| Formula weight | 382.41 |
| Temperature/K | 173.00 |
| Crystal system | orthorhombic |
| Space group | P2_1_2_1_2_1_ |
| a/Å | 6.85300(10) |
| b/Å | 14.5891(3) |
| c/Å | 18.5470(4) |
| α/° | 90 |
| β/° | 90 |
| γ/° | 90 |
| Volume/Å^3^ | 1854.31(6) |
| Z | 4 |
| ρ_calc_g/cm^3^ | 1.370 |
| μ/mm^‑1^ | 0.803 |
| F(000) | 808.0 |
| Crystal size/mm^3^ | 0.23 × 0.23 × 0.21 |
| Radiation | Cu Kα (λ = 1.54178) |
| 2Θ range for data collection/° | 9.536 to 136.804 |
| Index ranges | -5 ≤ h ≤ 8, -17 ≤ k ≤ 17, -22 ≤ l ≤ 21 |
| Reflections collected | 11772 |
| Independent reflections | 3352 [R_int_ = 0.0620, R_sigma_ = 0.0536] |
| Data/restraints/parameters | 3352/0/256 |
| Goodness-of-fit on F^2^ | 1.119 |
| Final R indexes [I>=2σ (I)] | R_1_ = 0.0562, wR_2_ = 0.1474 |
| Final R indexes [all data] | R_1_ = 0.0573, wR_2_ = 0.1501 |
| Largest diff. peak/hole / e Å^-3^ | 0.51/-0.66 |
| Flack parameter | -0.01(10) |

| Table S4 Crystal data and structure refinement for **Phen-2DICy** | |
| --- | --- |
| Identification code | **Phen-2DICy** |
| CCDC | 2445308 |
| Empirical formula | C_20_H_24_N_4_O_6_ |
| Formula weight | 416.43 |
| Temperature/K | 298.02 |
| Crystal system | monoclinic |
| Space group | P2_1_/c |
| a/Å | 11.927(3) |
| b/Å | 18.909(4) |
| c/Å | 8.6561(18) |
| α/° | 90 |
| β/° | 96.407(7) |
| γ/° | 90 |
| Volume/Å^3^ | 1940.0(7) |
| Z | 4 |
| ρ_calc_g/cm^3^ | 1.426 |
| μ/mm^‑1^ | 0.107 |
| F(000) | 880.0 |
| Crystal size/mm^3^ | 0.2 × 0.2 × 0.2 |
| Radiation | Mo Kα (λ = 0.71073) |
| 2Θ range for data collection/° | 4.308 to 56.708 |
| Index ranges | -15 ≤ h ≤ 15, -25 ≤ k ≤ 25, -10 ≤ l ≤ 11 |
| Reflections collected | 32321 |
| Independent reflections | 4848 [R_int_ = 0.1069, R_sigma_ = 0.0738] |
| Data/restraints/parameters | 4848/0/277 |
| Goodness-of-fit on F^2^ | 1.009 |
| Final R indexes [I>=2σ (I)] | R_1_ = 0.0509, wR_2_ = 0.1054 |
| Final R indexes [all data] | R_1_ = 0.1141, wR_2_ = 0.1287 |
| Largest diff. peak/hole / e Å^-3^ | 0.19/-0.26 |

| Table S5 Crystal data and structure refinement for **Phen-2DIC2SMe** | |
| --- | --- |
| Identification code | **Phen-2DIC2SMe** |
| CCDC | 2448447 |
| Empirical formula | C_20_H_22_N_4_O_2_S_2_ |
| Formula weight | 414.53 |
| Temperature/K | 173.00 |
| Crystal system | orthorhombic |
| Space group | Pbca |
| a/Å | 18.1126(5) |
| b/Å | 9.1955(2) |
| c/Å | 23.2813(6) |
| α/° | 90 |
| β/° | 90 |
| γ/° | 90 |
| Volume/Å^3^ | 3877.60(17) |
| Z | 8 |
| ρ_calc_g/cm^3^ | 1.420 |
| μ/mm^‑1^ | 2.693 |
| F(000) | 1744.0 |
| Crystal size/mm^3^ | 0.23 × 0.22 × 0.21 |
| Radiation | Cu Kα (λ = 1.54178) |
| 2Θ range for data collection/° | 7.594 to 136.6 |
| Index ranges | -21 ≤ h ≤ 21, -11 ≤ k ≤ 11, -28 ≤ l ≤ 28 |
| Reflections collected | 56406 |
| Independent reflections | 3547 [R_int_ = 0.0800, R_sigma_ = 0.0319] |
| Data/restraints/parameters | 3547/0/256 |
| Goodness-of-fit on F^2^ | 1.063 |
| Final R indexes [I>=2σ (I)] | R_1_ = 0.0350, wR_2_ = 0.0941 |
| Final R indexes [all data] | R_1_ = 0.0398, wR_2_ = 0.0977 |
| Largest diff. peak/hole / e Å^-3^ | 0.31/-0.31 |

Figure S9 ^1^H NMR spectrum of **Phen-2DICy** in CDCl_3_*.*

Figure S10 Comparison of ^1^H NMR spectrum of **Phen-2DIC2OMe** and **Phen-2DICy** in CDCl_3_*.* The shaded blue area indicated the most obvious chemical shifts region.

Figure S11 Comparison of ^1^H NMR spectrum of **Phen-2DIC2OMe** and **Phen-2DIC2SMe** in CDCl_3_*.* The sizes of the blue circles represent the relative atomic radii. The dashed blue line and the arrows were used to show the peak shifts.

Figure S12 Comparison of ^13^C NMR spectrum of **Phen-2DIC2OMe** and **Phen-2DIC2SMe** in CDCl_3_*.*


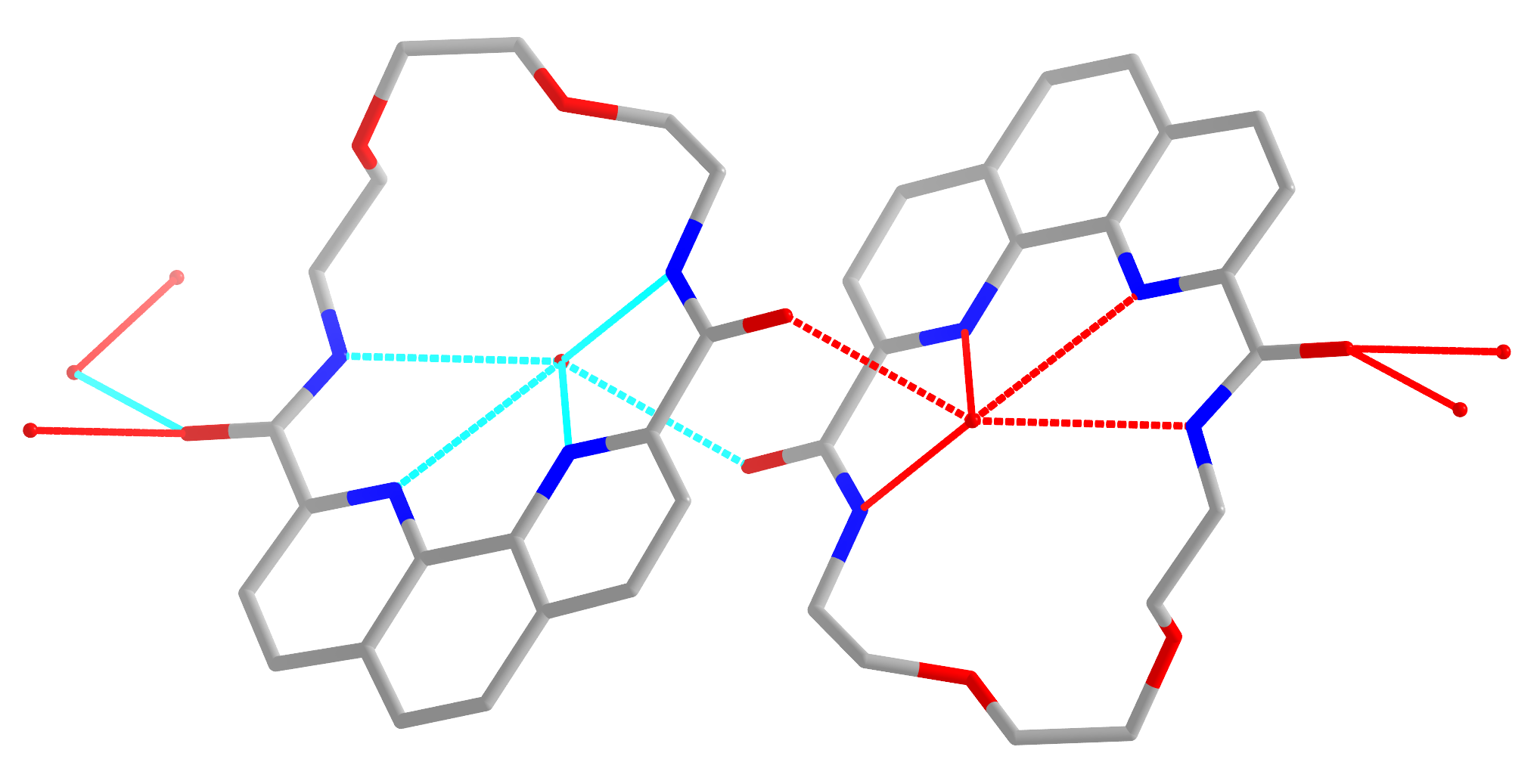


Figure S13 Water mediate hydrogen bond network in **Phen-2DICy**.


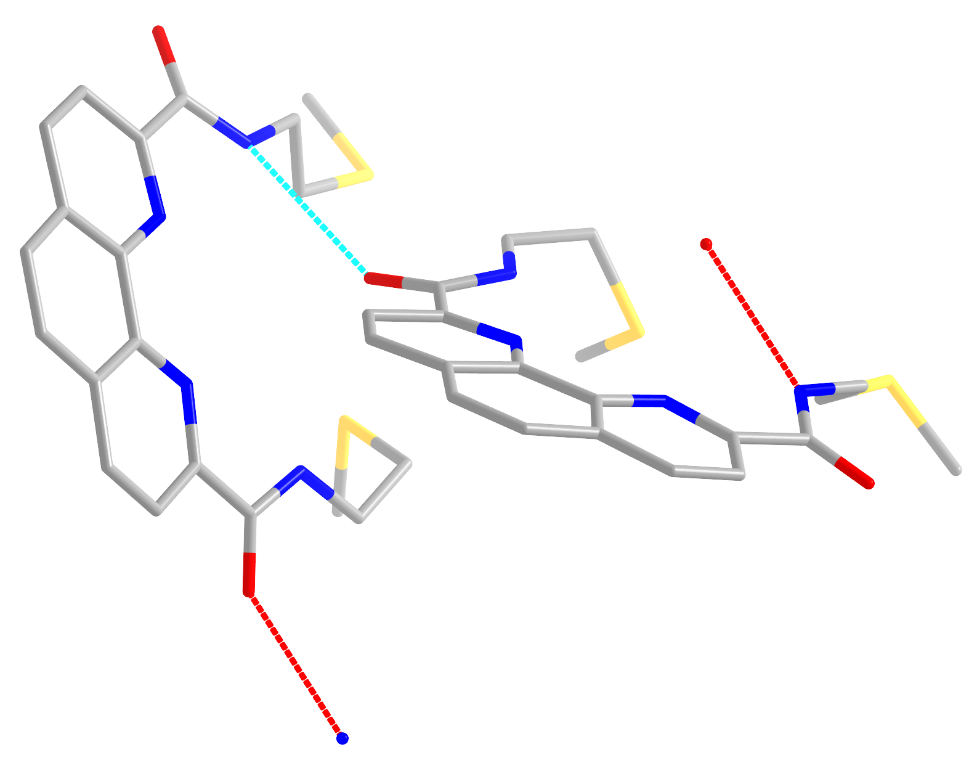


Figure S14 Intermolecular hydrogen bond network in **Phen-2DIC2SMe**.


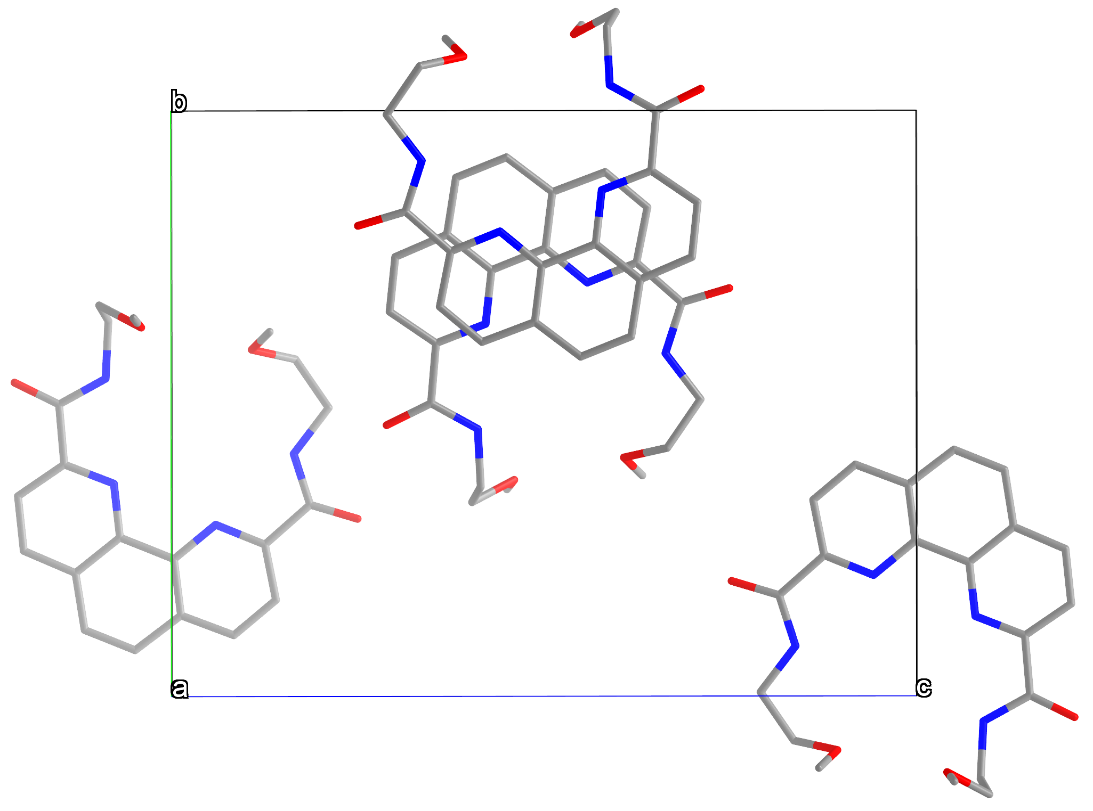


Figure S15 Crystal structures for **Phen-2DIC2OMe** in one unit cell viewing along a-axis.

Figure S16 Emission spectra (left) and normalized absorption spectra (right) for **Phen-2DIC2OMe** (black trace), **Phen-2DICy** (red trace) and **Phen-2DIC2SMe** (blue trace) in methanol with concentrations of 2×10^-5^ M. Excitation wavelengths for emission spectra were 320 nm.


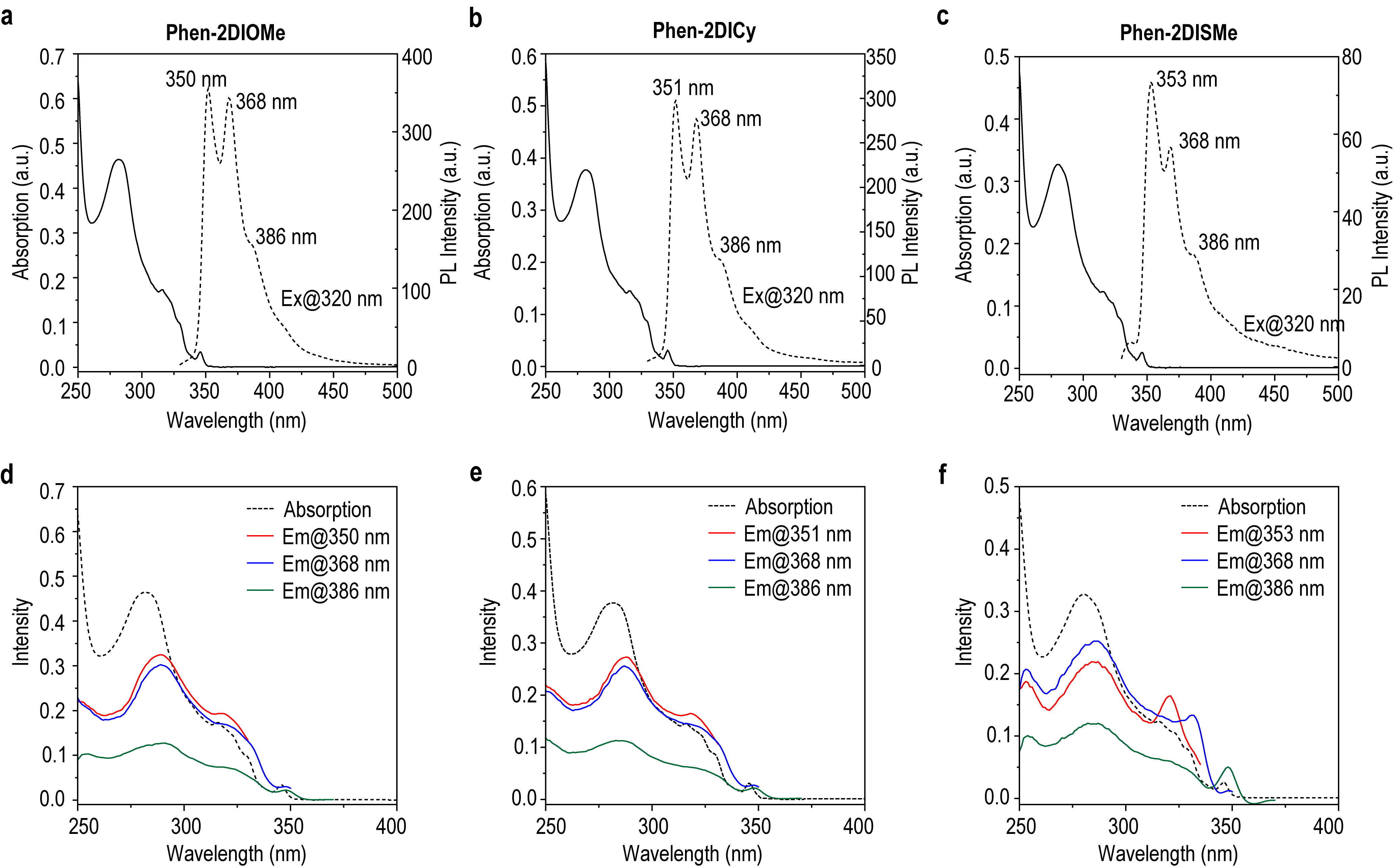


Figure S17 Absorption spectra (solid line) and emission spectra (dash line) (a) and the corresponding excitation spectra at each emission peaks (d) for **Phen-2DIC2OMe**, **Phen-2DICy** (b and e) and **Phen-2DIC2SMe** (c and f) in methanol with concentrations of 2×10^-5^ M. Excitation wavelengths for emission spectra were given in the figures.

Figure S18 Cyclic voltammetry scans for **Phen-2DIC2OMe** (black trace), **Phen-2DICy** (red trace) and **Phen-2DIC2SMe** (blue trace) in dry DMF with ligand concentrations of 1 mM. at the present of 0.2 M of Bu_4_NF_6_ as supporting electrolyte. The scan rate was 100 mV/s.

Figure S19 Detailed solubility tests for **Phen-2DIC2OMe** (top), **Phen-2DICy** (middle) and **Phen-2DIC2SMe** (bottom) in 1 M HNO_3_ and octanol. S, P and PS represented soluble, precipitated and partially soluble. The final solubilities were summarized.


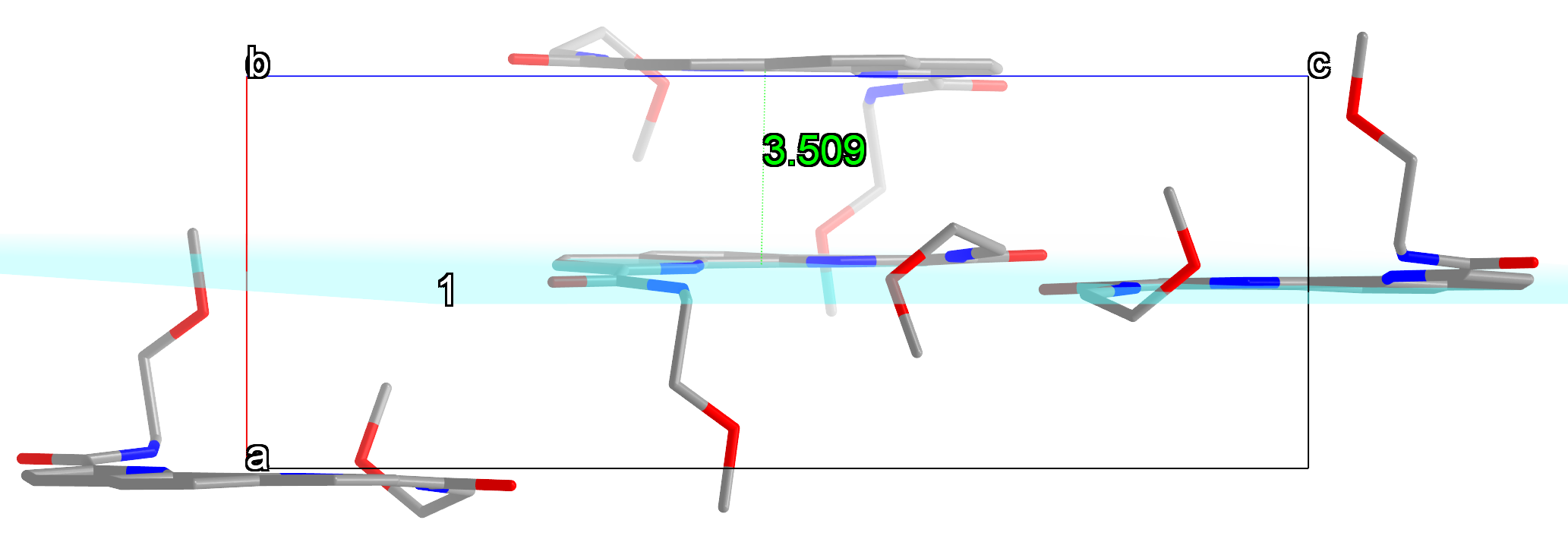


Figure S20 Crystal structures for **Phen-2DIC2OMe** showing the π-π stacking of two molecules in one unit cell viewing along b-axis. One of the phenanthroline plane was defined as plane 1. A distance of 3.509 Å was measured.


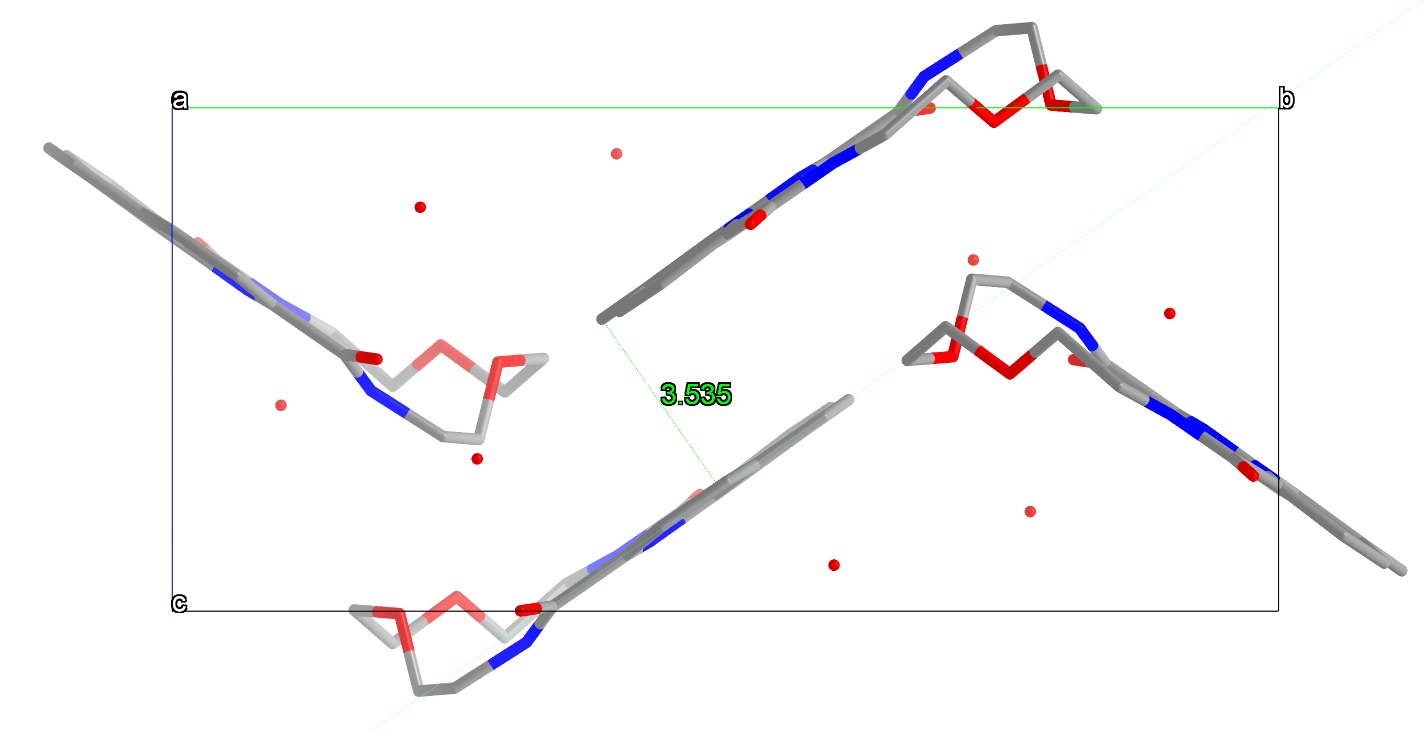


Figure S21 Crystal structures for **Phen-2DICy** showing the π-π stacking of two molecules in one unit cell viewing along a-axis. A distance of 3.535 Å was measured.

Table S6 Separation performance comparisons of literature reported hydrophilic masking ligands with liquid-liquid-based extraction

| *SF*_Eu(Ⅲ)/Am(Ⅲ)_ | Experimental conditions | References |
| --- | --- | --- |
| ~47 | ^[a]^O: 0.2 M **TODGA** in 5% 1-octanol/kerosene;  ^[b]^A: 10 mM **BTrzPhen** in 0.33 M HNO_3_ | [3] |
| ~30 | O: 0.1 M **TODGA** in 5% 1-octanol/TPH (a French kerosene);  A: 20 mM **EtOH-BPTD** in 0.5 M HNO_3_ | [4] |
| ~170 | O: 0.03 M **TODGA** in kerosene;  A: 1 mM **DS-Ph-DAPhen** in ca. 0.3 M HNO_3_ containing 1 M NaNO_3_ | [5] |
| ~60 | O: 0.2 M **TODGA** in 5% 1-octanol/kerosene;  A: 10 mM **TS-BTPhen2** in ca. 1.0 M HNO_3_ | [6] |
| ~140 | O: 0.2 M **TODGA** in 5% 1-octanol/kerosene;  A: 100 mM **PyTri** in ca. 0.25 M HNO_3_ | [7] |
| ~12 | O: 0.3 M **HDEHP**, 0.6 M **DMDOHEMA** in **TPH**;  A: 0.5 M **HEDTA** at pH = 3 | [8] |
| ~11 | O: 50 mM **NTAamide** (*n*-Oct) in dodecane;  A: 20 mM **TEE-BisDGA** at pH = 3 | [9] |
| ~34 | O: 50 mM **NTAamide** (*n*-Oct) in dodecane;  A: 20 mM **SO3-TEX(m)-BisDGA** at pH = 3 | [10] |
| ~30 | O: 20 mM **TODGA** with 5% (vol) octanol in **TPH**;  A: 50 mM **PrOH-BPTD** in 0.5 M HNO_3_ | [4] |
| ~40 | O: 50 mM **TODGA** with 5% (vol) octanol in kerosene;  A: 10 mM ligand in 1 M HNO_3_ with 0.5 M NH_4_NO_3_ | [11] |

^[a]^ Organic phase. ^[b]^ Aqueous phase.

Figure S22. Distribution ratios (*D*_M_, bar graphs) and separation factors (*SF*, boxes) obtained in the extraction of Am(III) and Eu(III) by **TODGA** with **Phen-2DIC2OMe** as functions of NaNO_3_ concentrations. Extraction conditions: organic phase (O): 100 mM **TODGA** in dodecane; aqueous phase (A): 10 mM **Phen-2DIC2OMe** in 0.75 M HNO_3_ with different concentrations of NaNO_3_. O/A = 1; Vortex shaker (50 Hz) for 30 minutes at 25 ℃. Error bars were averaged from three duplicated experiments.

Figure S23. Distribution ratios (*D*_M_, bar graphs) and separation factors (*SF*, boxes) obtained in the extraction of Am(III) and Eu(III) by **TODGA** with **Phen-2DICy** as functions of NaNO_3_ concentrations. Extraction conditions: organic phase (O): 100 mM **TODGA** in dodecane; aqueous phase (A): 10 mM **Phen-2DICy** in 0.75 M HNO_3_ with different concentrations of NaNO_3_. O/A = 1; Vortex shaker (50 Hz) for 30 minutes at 25 ℃. Error bars were averaged from three duplicated experiments.

Figure S24 Distribution ratios (*D*_M_, bar graphs) and separation factors (*SF*, boxes) obtained in the extraction of Am(III) and Eu(III) by **TODGA** with **Phen-2DC2OMe** as functions of **Phen-2DIC2OMe** concentrations. Extraction conditions: organic phase (O): 100 mM **TODGA** in dodecane; aqueous phase (A): **Phen-2DIC2OMe** in 0.75 M HNO_3_ with various concentrations. O/A = 1; Vortex shaker (50 Hz) for 30 minutes at 25 ℃. Error bars were averaged from three duplicated experiments.

Figure S25 Distribution ratios (*D*_M_, bar graphs) and separation factors (*SF*, boxes) obtained in the extraction of Am(III) and Eu(III) by **TODGA** with **Phen-2DCy** as functions of **Phen-2DICy** concentrations. Extraction conditions: organic phase (O): 100 mM **TODGA** in dodecane; aqueous phase (A): **Phen-2DICy** in 0.75 M HNO_3_ with various concentrations. O/A = 1; Vortex shaker (50 Hz) for 30 minutes at 25 ℃. Error bars were averaged from three duplicated experiments.

Figure S26 Distribution ratios for both Eu(III) and Am(III) as functions of **Phen-2DC2OMe** in log-log scale deduced from Figure S24. Error bars were averaged from three duplicated experiments.

Figure S27 Distribution ratios for both Eu(III) and Am(III) as functions of **Phen-2DICy** in log-log scale deduced from Figure S25. Error bars were averaged from three duplicated experiments.

Figure S28 Distribution ratios (*D*_M_, bar graphs) and separation factors (*SF*, boxes) obtained in the extraction of Am(III) and Eu(III) by **TODGA** with **Phen-2DIC2OMe** as functions of extraction time. Extraction conditions: organic phase (O): 100 mM **TODGA** in dodecane; aqueous phase (A): 10 mM **Phen-2DIC2OMe** in 0.75 M HNO_3_. O/A = 1; Vortex shaker (50 Hz) for different extraction time at 25 ℃. Error bars were averaged from three duplicated experiments.

Figure S29 Distribution ratios (*D*_M_, bar graphs) and separation factors (*SF*, boxes) obtained in the extraction of Am(III) and Eu(III) by **TODGA** with **Phen-2DICy** as functions of extraction time. Extraction conditions: organic phase (O): 100 mM **TODGA** in dodecane; aqueous phase (A): 10 mM **Phen-2DICy** in 0.75 M HNO_3_. O/A = 1; Vortex shaker (50 Hz) for different extraction time at 25 ℃. Error bars were averaged from three duplicated experiments.


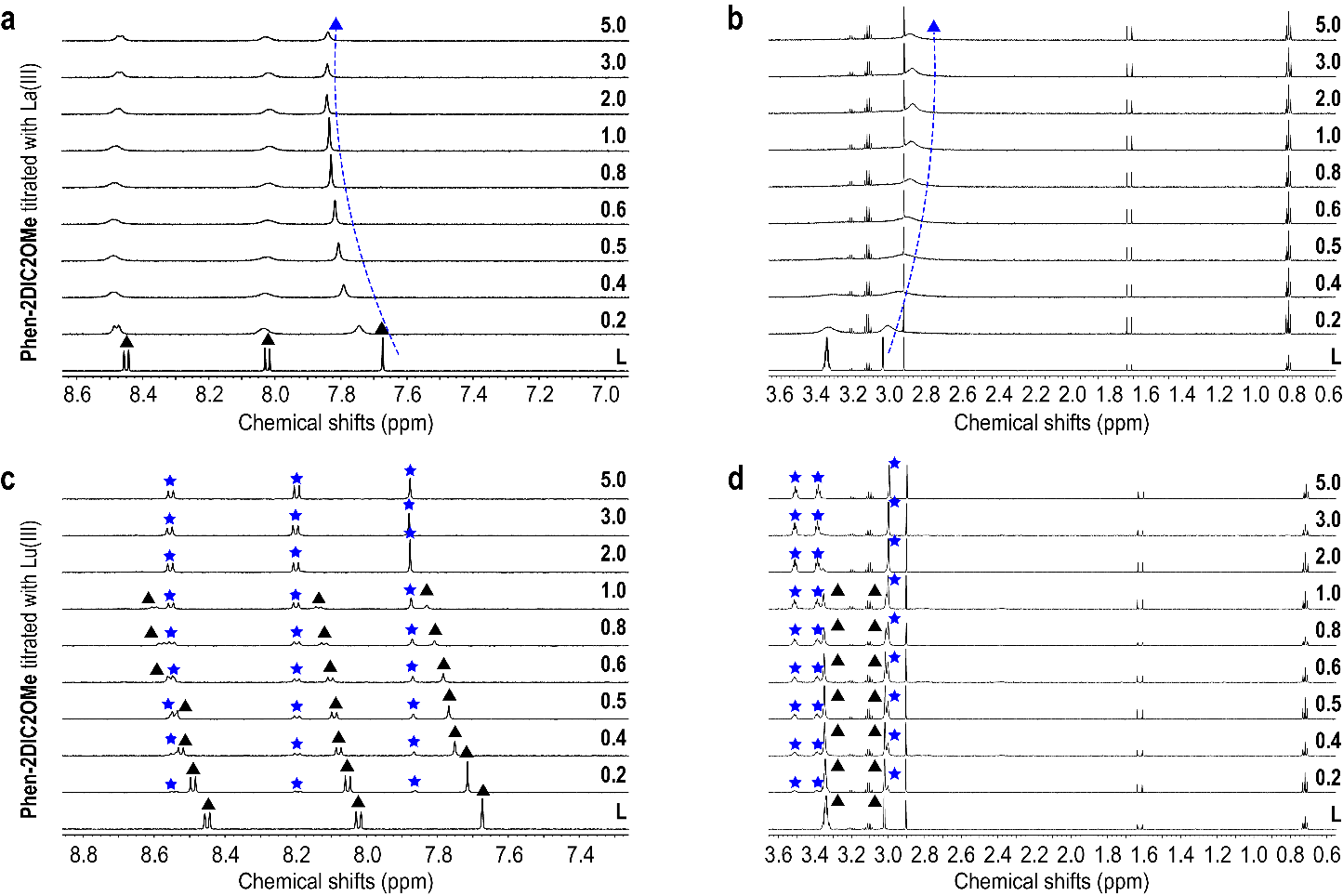


Figure S30 ^1^H NMR spectra of **Phen-2DIC2OMe** titrated with La(NO_3_)_3_ (a and b) and Lu(NO_3_)_3_ (c and d). Speculated species were marked as black triangles (ligand) and blue asterisks (metal/ligand of 1:1) respectively. Blue dashed arrows were used to show the chemical-shift changes during titrations. Experiments conditions: 10 mM **Phen-2DIC2OMe** in 0.75 M DNO_3_/D_2_O titrated with 0.1 M La(NO_3_)_3_ or Lu(NO_3_)_3_ at 25 ℃. The numbers on the right of the picture represent the molar ratio of the metal: the ligand.


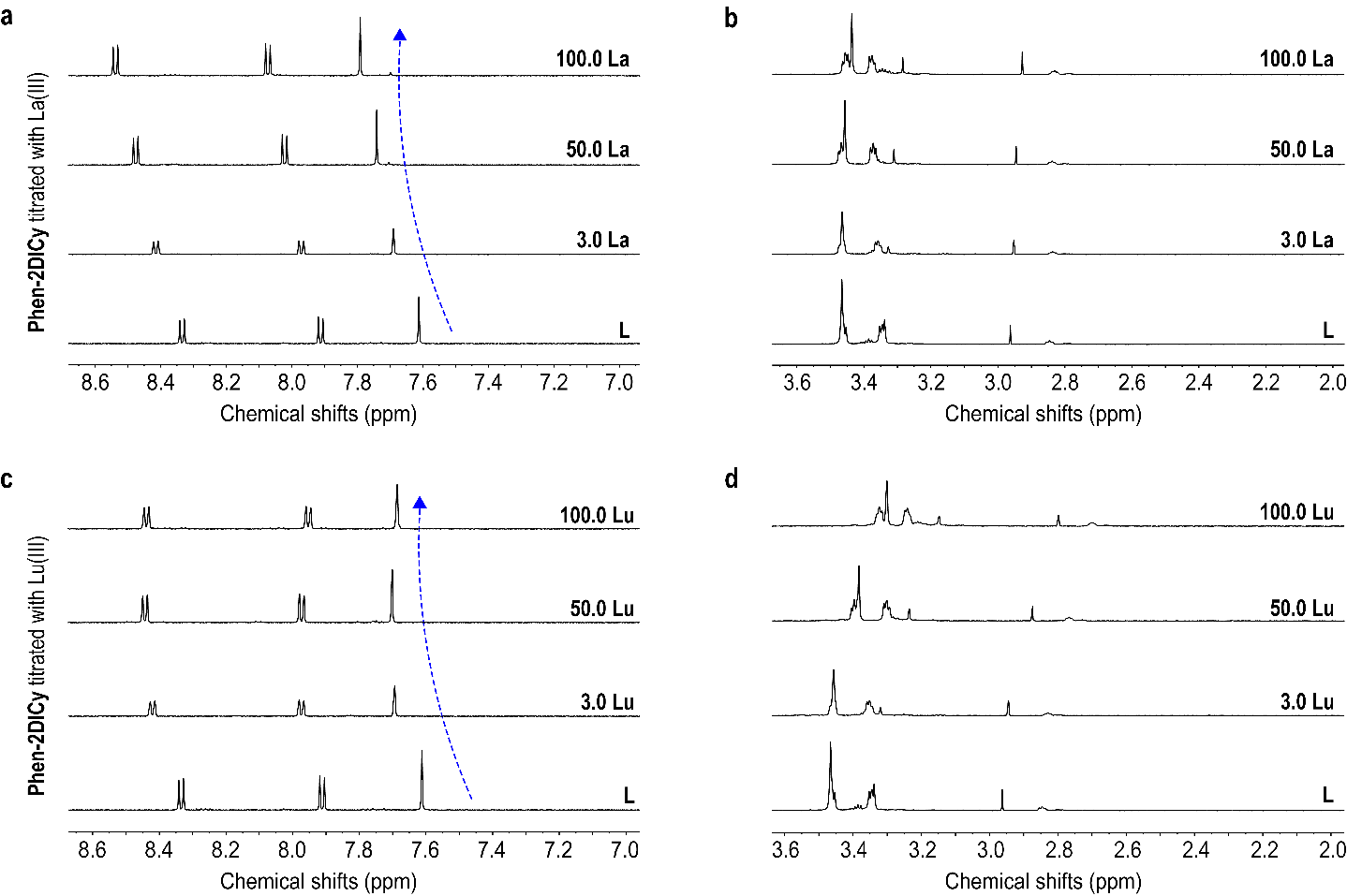


Figure S31 ^1^H NMR spectra of **Phen-2DICy** titrated with La(NO_3_)_3_ (a and b) and Lu(NO_3_)_3_ (c and d). Blue dashed arrows were used to show the chemical-shift changes during titrations. Experiments conditions: 10 mM **Phen-2DICy** in 0.75 M DNO_3_/D_2_O titrated with 0.1 M La(NO_3_)_3_ or Lu(NO_3_)_3_ at 25 ℃. The numbers on the right of the picture represent the molar ratio of the metal: the ligand.

Figure S32 Time dependent ^1^H NMR spectra of **Phen-2DICy** titrated with 100 equivalent of La(NO_3_)_3_ in 0.75 M DNO_3_/D_2_O excluding the possible slow coordination of **Phen-2DICy** to La(III).

Figure S33 Time dependent ^1^H NMR spectra of **Phen-2DICy** titrated with 100 equivalent of Lu(NO_3_)_3_ in 0.75 M DNO_3_/D_2_O excluding the possible slow coordination of **Phen-2DICy** to Lu(III).


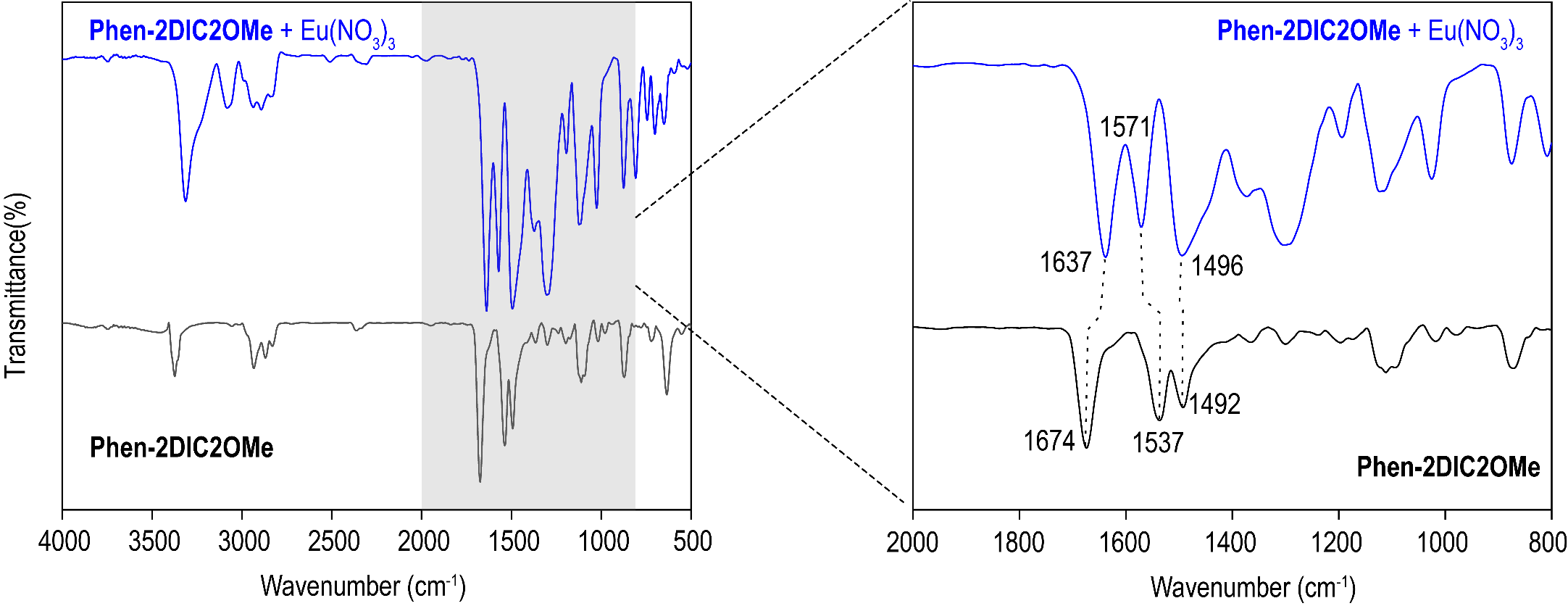


Figure S34 IR spectra for **Phen-2DIC2OMe** and **Phen-2DIC2OMe**-Eu (1:1). The right panel was zoomed-in from the gray region in the left panel.


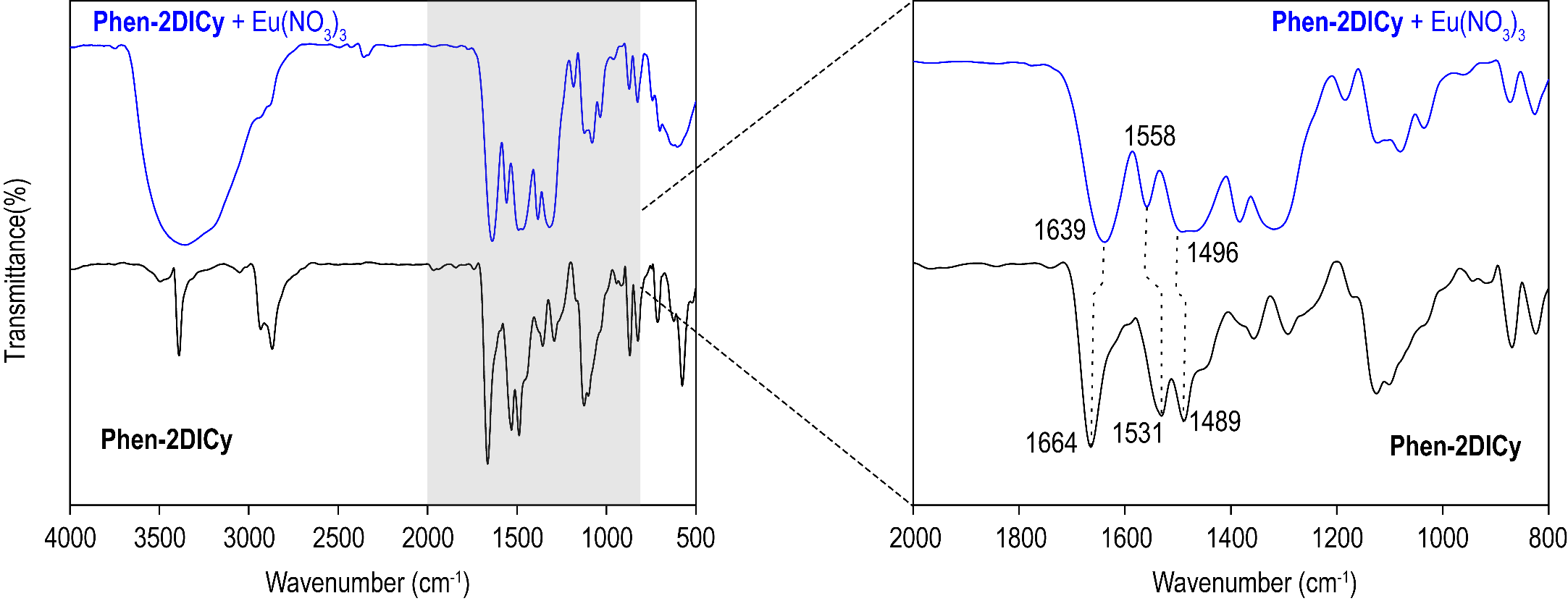


Figure S35 IR spectra for **Phen-2DICy** and **Phen-2DICy**-Eu (1:1). The right panel was zoomed-in from the gray region in the left panel.

Figure S36 Comparisons of IR spectra at the carbonyl regions for **Phen-2DIC2OMe**/**Phen-2DIC2OMe**-Eu (1:1) and **Phen-2DICy**/**Phen-2DICy**-Eu (1:1). The dashed lines are Gaussian fitting results with FWHM given on the figures.

Note S1

The quenching effect of -OH vibrations on metal-centered emission enables the fluorescence lifetime of the Eu(III) ^5^*D*_0_→^7^*F*_2_ transition to serve as an indicator of the number of water molecules (*N*_H2O_) within the first coordination sphere of Eu(III).[12] A well-established correlation between the *N*_H2O_ and the emission decay lifetime (τ, in µs) as expressed by the equation: *N*_H2O_ = 1.05 / τ – 0.7, which has an inherent uncertainty of ±0.5 water molecules.[13] The decay lifetimes (τ) used in this analysis resulted from fitting the corresponding decay profiles: R(t) = B_1_exp (-t/τ_1_) + B_2_exp (-t/τ_2_) + B_3_exp (-t/τ_3_).

TRLFS titrations for this study were performed in 0.75 M HNO₃ solution. This acid medium was selected because: (1) It gave the best separation efficiency for Eu(III)/Am(III), necessitating identical conditions for TRLFS to accurately simulate the extraction processes; (2) HNO_3_ was used with respect to the noncoordinating HClO_4_ because **Phen-2DIC2OMe**–Eu(Ⅲ) displayed markedly poor solubility in 0.7 M HClO_4_. As nitrate ions are known to be weak coordinating ligands, the number of inner-sphere water molecules calculated from the lifetime data (Figure 3c, f, blue data points) in the absence of added ligand was ca. 7.5, which is approximately 1.5 less than the commonly reported nine coordinated waters in similar systems,[14] suggesting the coordination of one nitrate anion to the metal center.


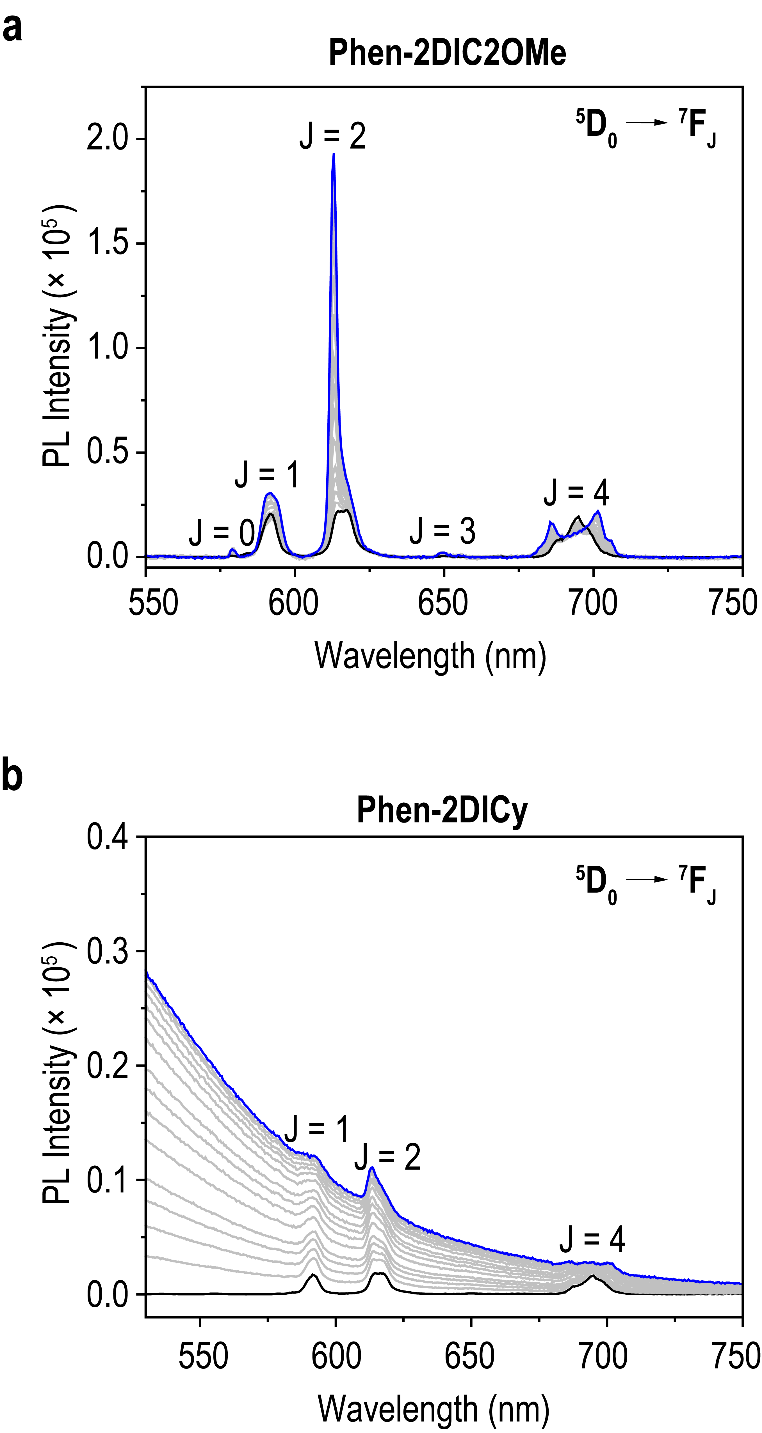


Figure S37 Emission from the Eu(III) characterized peaks during the TRLFS titrations of **Phen-2DIC2OMe** and **Phen-2DICy** towards Eu(III). Experiment condition: C_Ligand_/C_Eu(III)_ = 10 mM/1 mM in 0.75 M HNO_3_ with 1.0 M NaNO_3_. Totally 2.0 mL of titrant was added to V_0_ = 1.6 mL of initial solution.

Figure S38 Single crystal data comparisons of **Phen-2DIC2OMe**, **Phen-2DIC2SMe** and **DIPhen-C4** with Eu(III). The bond lengths were given on each complex. All single crystals were prepared under similar conditions of slow evaporating of the mixture of ligand/Eu(NO_3_)_3_ (1/1) mixture from the acholic solutions. 10 coordinated Eu(III) were observed for all three complexes with overall ligand/metal ratios of 1 for **Phen-2DIC2OMe** (a) and **DIPhen-C4** (c) while 2 for **Phen-2DIC2SMe** (b).

Table S7 Bond lengths summary for the complexes in Figure S38.

| Ligand | Eu-N (Å) | Average (Å) | Eu-O (Å) | Average (Å) |
| --- | --- | --- | --- | --- |
| **Phen-2DIC2OMe**-Eu | 2.613 | **2.611** | 2.405 | **2.397** |
|  | 2.610 |  | 2.388 |  |
| **Phen-2DIC2SMe**-Eu | 2.572 | **2.593** | 2.498 | **2.479** |
|  | 2.613 |  | 2.460 |  |
| **DIPhen-C4**-Eu | 2.587 | **2.589** | 2.422 | **2.411** |
|  | 2.591 |  | 2.400 |  |

Table S8 Crystal data and structure refinement for **Phen-2DIC2OMe**-Eu

| Identification code | **Phen-2DIC2OMe**-Eu |
| --- | --- |
| CCDC | 2448568 |
| Empirical formula | C_20_H_22_EuN_7_O_13_ |
| Formula weight | 720.40 |
| Temperature/K | 173.15 |
| Crystal system | orthorhombic |
| Space group | Pnn2 |
| a/Å | 15.6889(7) |
| b/Å | 18.8482(9) |
| c/Å | 8.7693(3) |
| α/° | 90 |
| β/° | 90 |
| γ/° | 90 |
| Volume/Å^3^ | 2593.15(19) |
| Z | 4 |
| ρ_calc_g/cm^3^ | 1.845 |
| μ/mm^‑1^ | 2.499 |
| F(000) | 1432.0 |
| Crystal size/mm^3^ | 0.2 × 0.2 × 0.2 |
| Radiation | Mo Kα (λ = 0.71073) |
| 2Θ range for data collection/° | 5.042 to 50.844 |
| Index ranges | -18 ≤ h ≤ 18, -22 ≤ k ≤ 22, -10 ≤ l ≤ 10 |
| Reflections collected | 43718 |
| Independent reflections | 4783 [R_int_ = 0.0557, R_sigma_ = 0.0341] |
| Data/restraints/parameters | 4783/41/373 |
| Goodness-of-fit on F^2^ | 1.064 |
| Final R indexes [I>=2σ (I)] | R_1_ = 0.0226, wR_2_ = 0.0486 |
| Final R indexes [all data] | R_1_ = 0.0255, wR_2_ = 0.0515 |
| Largest diff. peak/hole / e Å^-3^ | 0.61/-0.38 |
| Flack parameter | -0.003(14) |

Table S9 Crystal data and structure refinement for **Phen-2DIC2SMe**-Eu

| Identification code | **Phen-2DIC2SMe**-Eu |
| --- | --- |
| CCDC | 2448569 |
| Empirical formula | C_40_H_46_EuN_11_O_14_S_4_ |
| Formula weight | 1185.08 |
| Temperature/K | 299.00 |
| Crystal system | triclinic |
| Space group | P-1 |
| a/Å | 11.3982(6) |
| b/Å | 14.4833(6) |
| c/Å | 15.4530(8) |
| α/° | 105.500(2) |
| β/° | 102.028(2) |
| γ/° | 90.494(2) |
| Volume/Å^3^ | 2398.7(2) |
| Z | 2 |
| ρ_calc_g/cm^3^ | 1.641 |
| μ/mm^‑1^ | 1.558 |
| F(000) | 1204.0 |
| Crystal size/mm^3^ | 0.14 × 0.12 × 0.07 |
| Radiation | Mo Kα (λ = 0.71073) |
| 2Θ range for data collection/° | 4.528 to 53.042 |
| Index ranges | -14 ≤ h ≤ 14, -18 ≤ k ≤ 18, -19 ≤ l ≤ 19 |
| Reflections collected | 108386 |
| Independent reflections | 9914 [R_int_ = 0.0891, R_sigma_ = 0.0384] |
| Data/restraints/parameters | 9914/6/638 |
| Goodness-of-fit on F^2^ | 1.087 |
| Final R indexes [I>=2σ (I)] | R_1_ = 0.0449, wR_2_ = 0.1230 |
| Final R indexes [all data] | R_1_ = 0.0535, wR_2_ = 0.1301 |
| Largest diff. peak/hole / e Å^-3^ | 2.07/-1.63 |

Figure S39 ESP calculations for **Phen-2DIC2SMe** and **DIPhen-C4** showing the introduction of sulfur atoms perturb the electronic density on imine oxygens. The units for energy values are in kcal/mol.


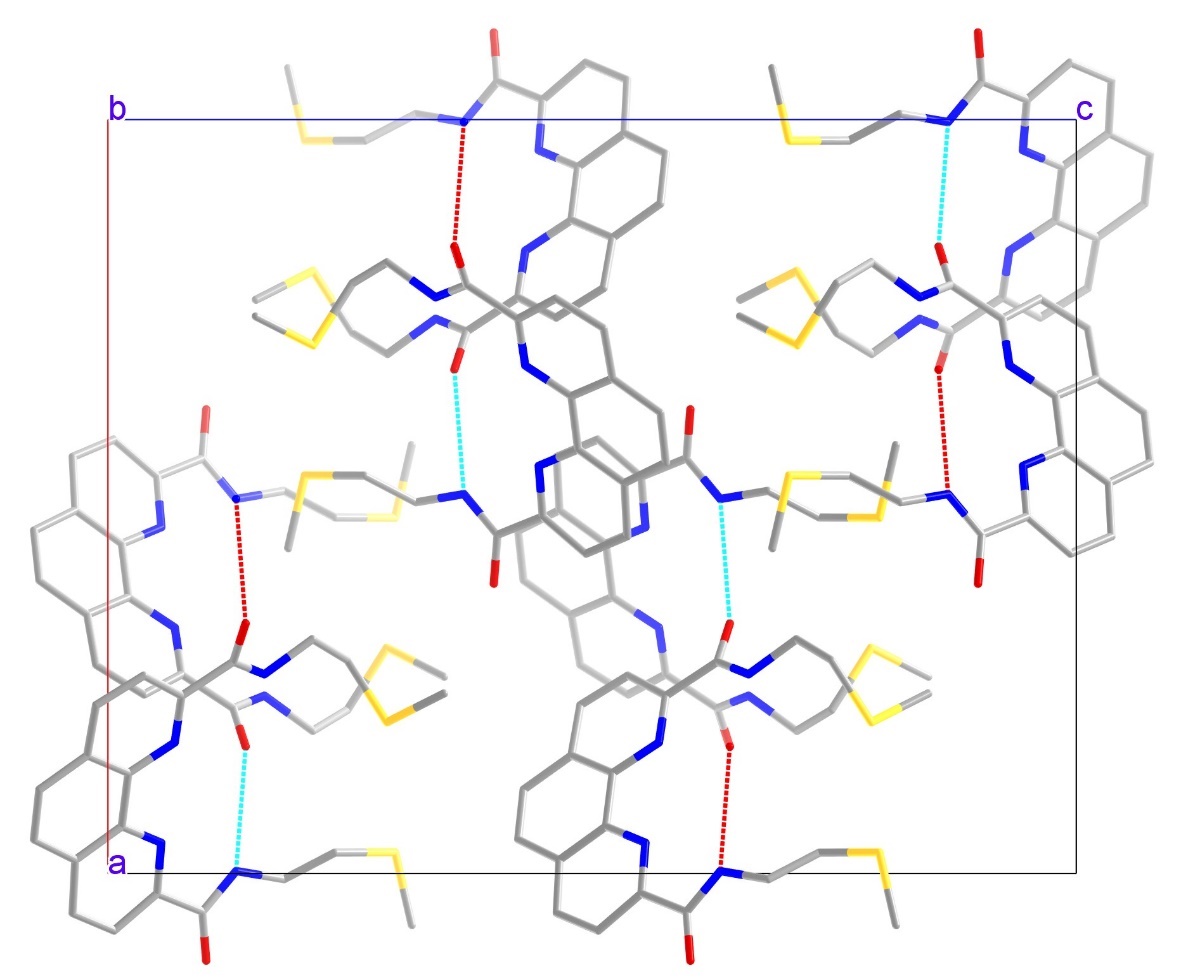


Figure S40 Hydrogen bonds in the unit cell of **Phen-2DIC2SMe**.

**SI references:**

[1] L. Duan, J. Fan, D. Tian, Q. Yan, X. Zhang, P. Li, C. Xu, L. Wang, Colloid Surf. A-Physicochem. Eng. Asp. 2022, 647, 129089, A novel and versatile precursor for the synthesis of highly preorganized tetradentate ligands based on phenanthroline and their binding properties towards lanthanides(III) ions.

[2] J. Figueiredo, I. Carreira-Barral, R. Quesada, J.-L. Mergny, C. Cruz, Bioorg. Med. Chem. 2022, 73, 116971, Synthesis and evaluation of 2,9-disubstituted-1,10-phenanthroline derivatives as G-quadruplex binders.

[3] A. C. Edwards, P. Mocilac, A. Geist, L. M. Harwood, C. A. Sharrad, N. A. Burton, R. C. Whitehead, M. A. Denecke, Chem. Commun. 2017, 53 (36), 5001, Hydrophilic 2,9-bis-triazolyl-1,10-phenanthroline ligands enable selective Am(iii) separation: a step further towards sustainable nuclear energy.

[4] P. Weßling, M. Maag, G. Baruth, T. Sittel, F. S. Sauerwein, A. Wilden, G. Modolo, A. Geist, P. J. Panak, Inorg. Chem. 2022, 61 (44), 17719, Complexation and Extraction Studies of Trivalent Actinides and Lanthanides with Water-Soluble and CHON-Compatible Ligands for the Selective Extraction of Americium.

[5] P. Ren, P.-w. Huang, X.-f. Yang, Y. Zou, W.-q. Tao, S.-l. Yang, Y.-h. Liu, Q.-y. Wu, L.-y. Yuan, Z.-f. Chai, W.-q. Shi, Inorg. Chem. 2021, 60 (1), 357, Hydrophilic Sulfonated 2,9-Diamide-1,10-phenanthroline Endowed with a Highly Effective Ligand for Separation of Americium(III) from Europium(III): Extraction, Spectroscopy, and Density Functional Theory Calculations.

[6] F. W. Lewis, L. M. Harwood, M. J. Hudson, A. Geist, V. N. Kozhevnikov, P. Distler, J. John, Chem. Sci. 2015, 6 (8), 4812, Hydrophilic sulfonated bis-1,2,4-triazine ligands are highly effective reagents for separating actinides(iii) from lanthanides(iii) via selective formation of aqueous actinide complexes.

[7] E. Macerata, E. Mossini, S. Scaravaggi, M. Mariani, A. Mele, W. Panzeri, N. Boubals, L. Berthon, M.-C. Charbonnel, F. Sansone, A. Arduini, A. Casnati, J. Am. Chem. Soc. 2016, 138 (23), 7232, Hydrophilic Clicked 2,6-Bis-triazolyl-pyridines Endowed with High Actinide Selectivity and Radiochemical Stability: Toward a Closed Nuclear Fuel Cycle.

[8] M. Heitzmann, C. Gateau, L. Chareyre, M. Miguirditchian, M.-C. Charbonnel, P. Delangle, New J. Chem. 2010, 34 (1), 108, Water-soluble tetrapodal N,O ligands incorporating soft N-heterocycles for the selective complexation of Am(iii) over Ln(iii).

[9] Z. Wang, S. Ding, X. Hu, S. Li, D. Su, L. Zhang, Y. Liu, Y. Jin, Sep. Purif. Technol. 2017, 181, 148, Selective extraction of americium(III) over europium(III) ions in nitric acid solution by NTAamide(C8) using a novel water-soluble bisdiglycolamide as a masking agent.

[10] L. He, X. Wang, Q. Li, X. Xiao, F. Li, F. Luo, Q. Pan, S. Ding, J. Environ. Chem. Eng. 2023, 11 (2), 109536, Novel water-soluble aromatic bisdiglycolamide masking agents for the separation of trivalent americium over lanthanides by NTAamide(n-Oct) extractant.

[11] S. Scaravaggi, E. Macerata, M. Galletta, E. Mossini, A. Casnati, M. Anselmi, F. Sansone, M. Mariani, J. Radioanal. Nucl. Chem. 2015, 303 (3), 1811, Hydrophilic 1,10-phenanthroline derivatives for selective Am(III) stripping into aqueous solutions.

[12] W. D. Horrocks, Jr., D. R. Sudnick, J. Am. Chem. Soc. 1979, 101 (2), 334, Lanthanide ion probes of structure in biology. Laser-induced luminescence decay constants provide a direct measure of the number of metal-coordinated water molecules.

[13] P. P. Barthelemy, G. R. Choppin, Inorg. Chem. 1989, 28 (17), 3354, Luminescence study of complexation of europium and dicarboxylic acids.

[14] a) D. Tian, Y. Liu, Y. Kang, Y. Zhao, P. Li, C. Xu, L. Wang, ACS Cent. Sci. 2023, 9 (8), 1642, A Simple yet Efficient Hydrophilic Phenanthroline-Based Ligand for Selective Am(III) Separation under High Acidity; b) Y. Liu, Y. Kang, M. Bao, H. Cao, C. Weng, X. Dong, H. Hao, X. Tang, J. Chen, L. Wang, C. Xu, J. Hazard. Mater. 2024, 462, 132756, Hydroxyl-group functionalized phenanthroline diimides as efficient masking agents for Am(III)/Eu(III) separation under harsh conditions.
