# Supplementary material for: On the Design of Effective Water‐Soluble Actinide‐Masking Ligands Through Ligand Structure Modulation
Source: Adv Sci (Weinh). 2025 Aug 21;12(42):e12292. doi: 10.1002/advs.202512292 (PMC12622539; doi:10.1002/advs.202512292)

checkCIF/PLATON report

Structure factors have been supplied for datablock(s) mo_b3_1

THIS REPORT IS FOR GUIDANCE ONLY. IF USED AS PART OF A REVIEW PROCEDURE FOR PUBLICATION, IT SHOULD NOT REPLACE THE EXPERTISE OF AN EXPERIENCED CRYSTALLOGRAPHIC REFEREE.

No syntax errors found.   CIF dictionary   Interpreting this report

Datablock: mo_b3_1

Bond precision:	C-C = 0.0076 A		Wavelength=0.71073
Cell:	a=11.3982(6)	b=14.4833(6)	c=15.4530(8)
	alpha=105.500(2)	beta=102.028(2)	gamma=90.494(2)
Temperature:	299 K		

	Calculated	Reported
Volume	2398.7(2)	2398.7(2)
Space group	P -1	P -1
Hall group	-P 1	-P 1
Moiety formula	C40 H44 Eu N9 O7 S4, 2(N O3), H2 O	C40 H44 Eu N9 O7 S4, 2(N O3), H2 O
Sum formula	C40 H46 Eu N11 O14 S4	C40 H46 Eu N11 O14 S4
Mr	1185.09	1185.08
Dx,g cm-3	1.641	1.641
Z	2	2
Mu (mm-1)	1.558	1.558
F000	1204.0	1204.0
F000'	1205.15	
h,k,lmax	14,18,19	14,18,19
Nref	9961	9914
Tmin,Tmax	0.804,0.897	0.679,0.745
Tmin'	0.804	

Correction method= # Reported T Limits: Tmin=0.679 Tmax=0.745
AbsCorr = MULTI-SCAN

Data completeness= 0.995   Theta(max)= 26.521

R(reflections)= 0.0449(8690)   wR2(reflections)= 0.1301(9914)

S = 1.087   Npar= 638

The following ALERTS were generated. Each ALERT has the format
       test-name_ALERT_alert-type_alert-level.
Click on the hyperlinks for more details of the test.

Alert level C

```
PLAT220_ALERT_2_C NonSolvent   Resd 1  C   Ueq(max)/Ueq(min) Range        5.0 Ratio
PLAT222_ALERT_3_C NonSolvent Resd 1  H   Uiso(max)/Uiso(min) Range        4.8 Ratio
PLAT234_ALERT_4_C Large Hirshfeld Difference S4       --C1       .       0.22 Ang.
PLAT234_ALERT_4_C Large Hirshfeld Difference N10      --C3       .       0.17 Ang.
PLAT242_ALERT_2_C Low    'MainMol' Ueq as Compared to Neighbors of         S2 Check
PLAT244_ALERT_4_C Low    'Solvent' Ueq as Compared to Neighbors of         N1 Check
PLAT244_ALERT_4_C Low    'Solvent' Ueq as Compared to Neighbors of         N2 Check
PLAT260_ALERT_2_C Large Average Ueq of Residue Including        O1      0.103 Check
PLAT260_ALERT_2_C Large Average Ueq of Residue Including        O4      0.124 Check
PLAT260_ALERT_2_C Large Average Ueq of Residue Including       O12      0.194 Check
PLAT910_ALERT_3_C Missing # of FCF Reflection(s) Below Theta(Min).          5 Note
                1  0  0,   0  1  0,   0 -1  1,  -1  0  1,   0  0  1,
PLAT911_ALERT_3_C Missing FCF Refl Between Thmin & STh/L=    0.600          6 Report
               -1  3  0,   3  4  0,   0 -3  1,   0  5  1,   1 -3  2,  -1 -2  2,
PLAT971_ALERT_2_C Check Calcd Resid. Dens.  1.49Ang From S3              2.17 eA-3
PLAT971_ALERT_2_C Check Calcd Resid. Dens.  1.91Ang From C3              1.59 eA-3
PLAT971_ALERT_2_C Check Calcd Resid. Dens.  1.11Ang From C20             1.58 eA-3
PLAT975_ALERT_2_C Check Calcd Resid. Dens.  0.52Ang From O12     .       0.44 eA-3
PLAT976_ALERT_2_C Check Calcd Resid. Dens.  0.58Ang From O12     .      -0.49 eA-3
```

Alert level G

```
PLAT003_ALERT_2_G Number of Uiso or Uij Restrained non-H Atoms ...          1 Report
PLAT007_ALERT_5_G Number of Unrefined Donor-H Atoms .............           6 Report
              H3    H6    H7    H10   H12A  H12B
PLAT154_ALERT_1_G The s.u.'s on the Cell Angles are Equal ..(Note)      0.002 Degree
PLAT186_ALERT_4_G The CIF-Embedded .res File Contains ISOR Records          1 Report
PLAT232_ALERT_2_G Hirshfeld Test Diff (M-X)  Eu1      --O8       .        5.5 s.u.
PLAT720_ALERT_4_G Number of Unusual/Non-Standard Labels .........           1 Note
              N00I
PLAT779_ALERT_4_G Suspect or Irrelevant (Bond) Angle(s) in CIF ...      41.60 Deg.
              O11  -C24  -EU1     1_555   1_555   1_555 ......     #  203 Check
PLAT790_ALERT_4_G Centre of Gravity not Within Unit Cell: Resd.  #          3 Note
              N O3
PLAT860_ALERT_3_G Number of Least-Squares Restraints ............           6 Note
PLAT912_ALERT_4_G Missing # of FCF Reflections Above STh/L=  0.600         36 Note
PLAT933_ALERT_2_G Number of HKL-OMIT Records in Embedded .res File          6 Note
               -1 -2  2,  -1  3  0,   0 -3  1,   0  5  1,   1 -3  2,   3  4  0,
PLAT969_ALERT_5_G The 'Henn et al.' R-Factor-gap value ..........        3.90 Note
              Predicted wR2: Based on SigI**2  3.34 or SHELX Weight 12.37
PLAT978_ALERT_2_G Number C-C Bonds with Positive Residual Density.          0 Info
```

   0 **ALERT level A** = Most likely a serious problem - resolve or explain
   0 **ALERT level B** = A potentially serious problem, consider carefully
  17 **ALERT level C** = Check. Ensure it is not caused by an omission or oversight
  13 **ALERT level G** = General information/check it is not something unexpected

   1 ALERT type 1 CIF construction/syntax error, inconsistent or missing data

```
14 ALERT type 2 Indicator that the structure model may be wrong or deficient
 4 ALERT type 3 Indicator that the structure quality may be low
 9 ALERT type 4 Improvement, methodology, query or suggestion
 2 ALERT type 5 Informative message, check
```

It is advisable to attempt to resolve as many as possible of the alerts in all categories. Often the minor alerts point to easily fixed oversights, errors and omissions in your CIF or refinement strategy, so attention to these fine details can be worthwhile. In order to resolve some of the more serious problems it may be necessary to carry out additional measurements or structure refinements. However, the purpose of your study may justify the reported deviations and the more serious of these should normally be commented upon in the discussion or experimental section of a paper or in the "special_details" fields of the CIF. checkCIF was carefully designed to identify outliers and unusual parameters, but every test has its limitations and alerts that are not important in a particular case may appear. Conversely, the absence of alerts does not guarantee there are no aspects of the results needing attention. It is up to the individual to critically assess their own results and, if necessary, seek expert advice.

Publication of your CIF in IUCr journals

A basic structural check has been run on your CIF. These basic checks will be run on all CIFs submitted for publication in IUCr journals (*Acta Crystallographica*, *Journal of Applied Crystallography*, *Journal of Synchrotron Radiation*); however, if you intend to submit to *Acta Crystallographica Section C* or *E* or *IUCrData*, you should make sure that full publication checks are run on the final version of your CIF prior to submission.

Publication of your CIF in other journals

Please refer to the *Notes for Authors* of the relevant journal for any special instructions relating to CIF submission.

PLATON version of 06/01/2024; check.def file version of 05/01/2024

Datablock mo_b3_1 - ellipsoid plot

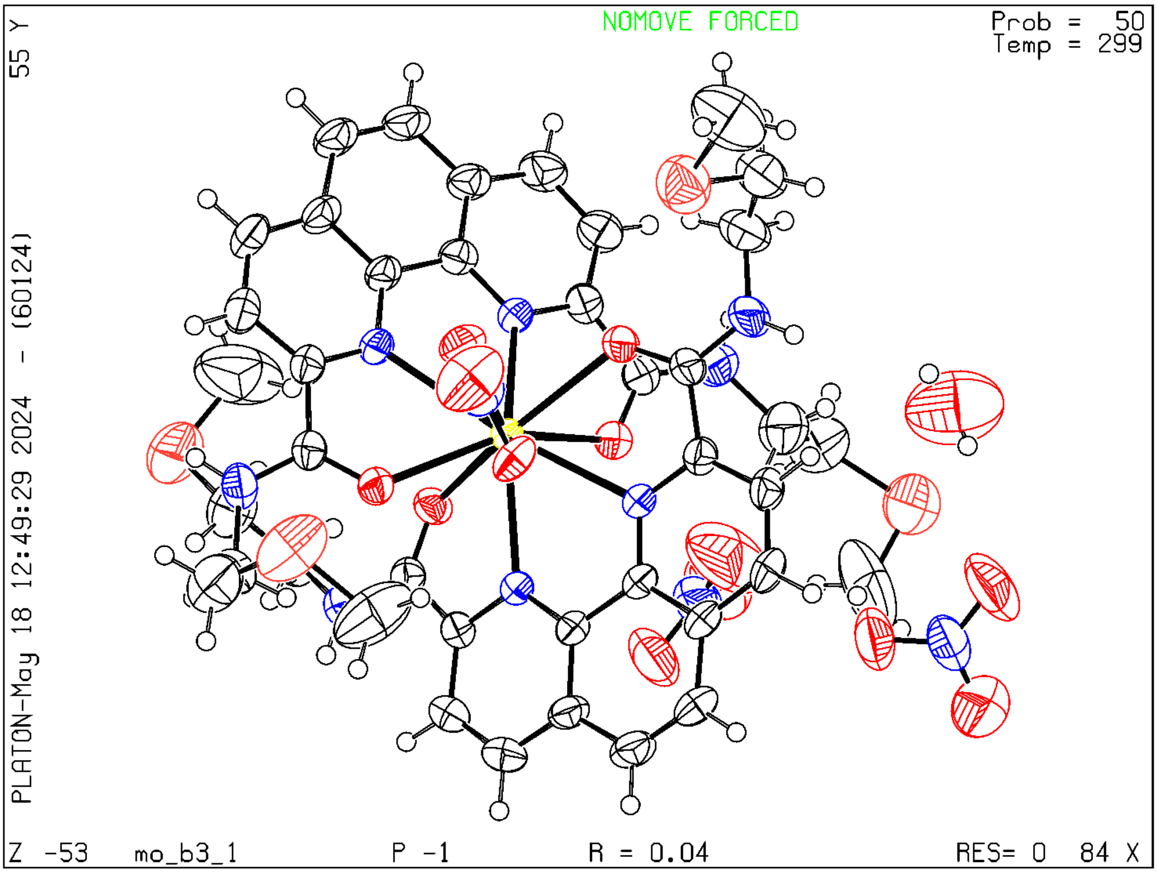

Supplement: Supplementary file 2 — Supporting Information [file ADVS-12-e12292-s001.zip › checkCIF_Phen-2DIC2SMe-Eu.pdf]
