# Supplementary material for: On the Design of Effective Water‐Soluble Actinide‐Masking Ligands Through Ligand Structure Modulation
Source: Adv Sci (Weinh). 2025 Aug 21;12(42):e12292. doi: 10.1002/advs.202512292 (PMC12622539; doi:10.1002/advs.202512292)

checkCIF/PLATON report

Structure factors have been supplied for datablock(s) aaa

THIS REPORT IS FOR GUIDANCE ONLY. IF USED AS PART OF A REVIEW PROCEDURE FOR PUBLICATION, IT SHOULD NOT REPLACE THE EXPERTISE OF AN EXPERIENCED CRYSTALLOGRAPHIC REFEREE.

No syntax errors found. CIF dictionary Interpreting this report

Datablock: aaa

Bond precision:	C-C = 0.0026 A		Wavelength=0.71073
Cell:	a=11.927(3)	b=18.909(4)	c=8.6561(18)
	alpha=90	beta=96.407(7)	gamma=90
Temperature:	298 K		

	Calculated	Reported
Volume	1940.0(8)	1940.0(7)
Space group	P 21/c	P 1 21/c 1
Hall group	-P 2ybc	-P 2ybc
Moiety formula	C20 H20 N4 O4, 2(H2 O)	2(H2 O), C20 H20 N4 O4
Sum formula	C20 H24 N4 O6	C20 H24 N4 O6
Mr	416.43	416.43
Dx,g cm-3	1.426	1.426
Z	4	4
Mu (mm-1)	0.107	0.107
F000	880.0	880.0
F000'	880.45	
h,k,lmax	15,25,11	15,25,11
Nref	4856	4848
Tmin,Tmax	0.979,0.979	0.562,0.746
Tmin'	0.979	

Correction method= # Reported T Limits: Tmin=0.562 Tmax=0.746
AbsCorr = MULTI-SCAN

Data completeness= 0.998   Theta(max)= 28.354

R(reflections)= 0.0509(2836)   wR2(reflections)= 0.1287(4848)

S = 1.009   Npar= 277

The following ALERTS were generated. Each ALERT has the format
test-name_ALERT_alert-type_alert-level.
Click on the hyperlinks for more details of the test.

Alert level C

```
PLAT042_ALERT_1_C Calc. and Reported MoietyFormula Strings  Differ     Please Check
              Calc: C20 H20 N4 O4, 2(H2 O)
              Rep.: 2(H2 O), C20 H20 N4 O4
PLAT417_ALERT_2_C Short Inter D-H..H-D       H1       ..H3B      .       2.14 Ang.
                                                   x,y,z  =      1_555 Check
PLAT790_ALERT_4_C Centre of Gravity not Within Unit Cell: Resd.  #          1 Note
              C20 H20 N4 O4
PLAT906_ALERT_3_C Large K Value in the Analysis of Variance ......      4.849 Check
PLAT911_ALERT_3_C Missing FCF Refl Between Thmin & STh/L=    0.600          2 Report
                0  2  2,   0  3  2,
```

Alert level G

```
PLAT007_ALERT_5_G Number of Unrefined Donor-H Atoms .............          6 Report
              H1    H4    H1A   H1B   H3A   H3B
PLAT910_ALERT_3_G Missing # of FCF Reflection(s) Below Theta(Min).          2 Note
                1  0  0,   1  1  0,
PLAT912_ALERT_4_G Missing # of FCF Reflections Above STh/L=  0.600          5 Note
PLAT913_ALERT_3_G Missing # of Very Strong Reflections in FCF ....          2 Note
                0  2  2,   0  3  2,
PLAT969_ALERT_5_G The 'Henn et al.' R-Factor-gap value ..........       1.90 Note
              Predicted wR2: Based on SigI**2  6.77 or SHELX Weight 13.14
PLAT978_ALERT_2_G Number C-C Bonds with Positive Residual Density.          4 Info
```

0 **ALERT level A** = Most likely a serious problem - resolve or explain
0 **ALERT level B** = A potentially serious problem, consider carefully
5 **ALERT level C** = Check. Ensure it is not caused by an omission or oversight
6 **ALERT level G** = General information/check it is not something unexpected

1 ALERT type 1 CIF construction/syntax error, inconsistent or missing data
2 ALERT type 2 Indicator that the structure model may be wrong or deficient
4 ALERT type 3 Indicator that the structure quality may be low
2 ALERT type 4 Improvement, methodology, query or suggestion
2 ALERT type 5 Informative message, check

It is advisable to attempt to resolve as many as possible of the alerts in all categories. Often the minor alerts point to easily fixed oversights, errors and omissions in your CIF or refinement strategy, so attention to these fine details can be worthwhile. In order to resolve some of the more serious problems it may be necessary to carry out additional measurements or structure refinements. However, the purpose of your study may justify the reported deviations and the more serious of these should normally be commented upon in the discussion or experimental section of a paper or in the "special_details" fields of the CIF. checkCIF was carefully designed to identify outliers and unusual parameters, but every test has its limitations and alerts that are not important in a particular case may appear. Conversely, the absence of alerts does not guarantee there are no aspects of the results needing attention. It is up to the individual to critically assess their own results and, if necessary, seek expert advice.

Publication of your CIF in IUCr journals

A basic structural check has been run on your CIF. These basic checks will be run on all CIFs submitted for publication in IUCr journals (*Acta Crystallographica*, *Journal of Applied Crystallography*, *Journal of Synchrotron Radiation*); however, if you intend to submit to *Acta Crystallographica Section C* or *E* or *IUCrData*, you should make sure that full publication checks are run on the final version of your CIF prior to submission.

Publication of your CIF in other journals

Please refer to the *Notes for Authors* of the relevant journal for any special instructions relating to CIF submission.

PLATON version of 13/12/2023; check.def file version of 13/12/2023

Datablock aaa - ellipsoid plot

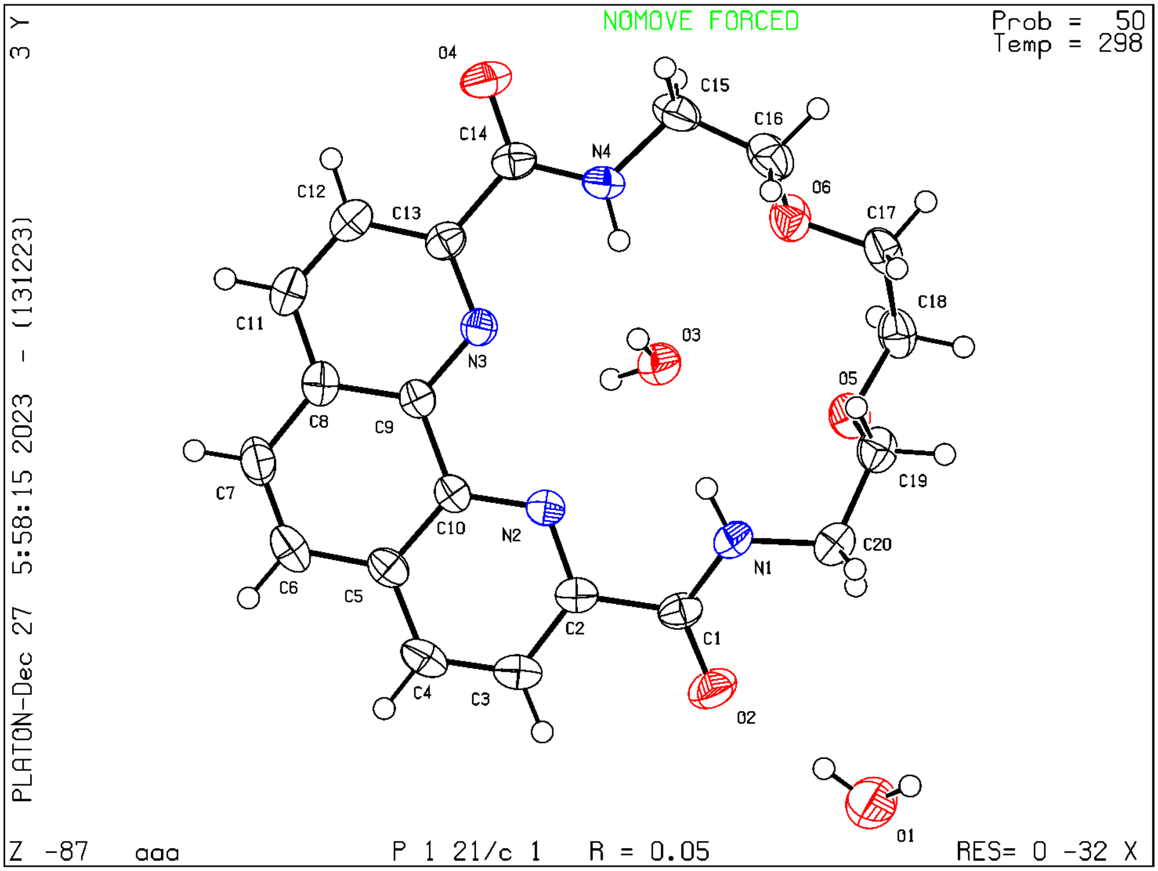

Supplement: Supplementary file 2 — Supporting Information [file ADVS-12-e12292-s001.zip › checkCIF_Phen-2DICy.pdf]
