# Supplementary material for: On the Design of Effective Water‐Soluble Actinide‐Masking Ligands Through Ligand Structure Modulation
Source: Adv Sci (Weinh). 2025 Aug 21;12(42):e12292. doi: 10.1002/advs.202512292 (PMC12622539; doi:10.1002/advs.202512292)
Supplement: Supplementary file 2 — Supporting Information [file ADVS-12-e12292-s001.zip › checkCIF_Phen-2DIC2OMe.pdf]

checkCIF (basic structural check) running

Checking for embedded fcf data in CIF ...
Found embedded fcf data in CIF. Extracting fcf data from uploaded CIF, please wait . . .

checkCIF/PLATON (basic structural check)

Structure factors have been supplied for datablock(s) ky31

THIS REPORT IS FOR GUIDANCE ONLY. IF USED AS PART OF A REVIEW PROCEDURE FOR PUBLICATION, IT SHOULD NOT REPLACE THE EXPERTISE OF AN EXPERIENCED CRYSTALLOGRAPHIC REFEREE.

No syntax errors found. CIF dictionary
Please wait while processing .... Interpreting this report

Structure factor report

Datablock: ky31

Bond precision:	C-C = 0.0034 A		Wavelength=1.54178
Cell:	a=6.8530(1)	b=14.5891(3)	c=18.5470(4)
	alpha=90	beta=90	gamma=90
Temperature:	173 K		

	Calculated	Reported
Volume	1854.31(6)	1854.31(6)
Space group	P 21 21 21	P 21 21 21
Hall group	P 2ac 2ab	P 2ac 2ab
Moiety formula	C20 H22 N4 O4	C20 H22 N4 O4
Sum formula	C20 H22 N4 O4	C20 H22 N4 O4
Mr	382.42	382.41
Dx,g cm-3	1.370	1.370
Z	4	4
Mu (mm-1)	0.803	0.803
F000	808.0	808.0
F000'	810.57	
h,k,lmax	8,17,22	8,17,22
Nref	3424[1986]	3352
Tmin,Tmax	0.831,0.845	0.438,0.753
Tmin'	0.831	

Correction method= # Reported T Limits: Tmin=0.438 Tmax=0.753 AbsCorr = MULTI-SCAN

Data completeness= 1.69/0.98 Theta(max)= 68.402

R(reflections)= 0.0562(3254) wR2(reflections)= 0.1501(3352)

S = 1.119 Npar= 256

The following ALERTS were generated. Each ALERT has the format
test-name_ALERT_alert-type_alert-level.
Click on the hyperlinks for more details of the test.

Alert level C

DIFMN02_ALERT_2_C The minimum difference density is < -0.1*ZMAX*0.75
_refine_diff_density_min given = -0.655
Test value = -0.600
DIFMN03_ALERT_1_C The minimum difference density is < -0.1*ZMAX*0.75
The relevant atom site should be identified.
PLAT098_ALERT_2_C Large Reported Min. (Negative) Residual Density -0.65 eA-3
PLAT911_ALERT_3_C Missing FCF Refl Between Thmin & STh/L= 0.600 34 Report
2 0 0, 6 1 0, 7 1 0, 8 1 0, 6 2 0, 7 3 0,
0 14 0, 7 0 1, 8 0 1, 7 1 1, 8 1 1, 7 1 2,
7 3 2, 4 0 3, 7 2 3, 0 3 3, 0 5 4, 0 11 4,
0 4 5, 1 4 5, 0 5 5, 1 5 5, 0 6 5, 2 6 5,
0 7 5, 1 7 5, 0 4 6, 1 5 6, 0 6 6, 1 6 6,

0 8 6, 0 7 8, 2 13 12, 2 10 16,
PLAT977_ALERT_2_C Check Negative Difference Density on H4 . -0.31 eA-3

Alert level G

PLAT007_ALERT_5_G Number of Unrefined Donor-H Atoms .... 2 Report
H1 H4
PLAT072_ALERT_2_G SHELXL First Parameter in WGHT Unusually Large 0.12 Report
PLAT333_ALERT_2_G Large Aver C6-Ring C-C Dist C8 -C13 . 1.42 Ang.
PLAT910_ALERT_3_G Missing # of FCF Reflection(s) Below Theta(Min). 1 Note
0 1 1,
PLAT912_ALERT_4_G Missing # of FCF Reflections Above STh/L= 0.600 1 Note
PLAT913_ALERT_3_G Missing # of Very Strong Reflections in FCF .... 1 Note
2 0 0,
PLAT969_ALERT_5_G The 'Henn et al.' R-Factor-gap value .... 2.706 Note
Predicted wR2: Based on SigI**2 5.55 or SHELX Weight 13.42
PLAT978_ALERT_2_G Number C-C Bonds with Positive Residual Density. 0 Info

0 **ALERT level A** = Most likely a serious problem - resolve or explain
0 **ALERT level B** = A potentially serious problem, consider carefully
5 **ALERT level C** = Check. Ensure it is not caused by an omission or oversight
8 **ALERT level G** = General information/check it is not something unexpected

1 ALERT type 1 CIF construction/syntax error, inconsistent or missing data
6 ALERT type 2 Indicator that the structure model may be wrong or deficient
3 ALERT type 3 Indicator that the structure quality may be low
1 ALERT type 4 Improvement, methodology, query or suggestion
2 ALERT type 5 Informative message, check

It is advisable to attempt to resolve as many as possible of the alerts in all categories. Often the minor alerts point to easily fixed oversights, errors and omissions in your CIF or refinement strategy, so attention to these fine details can be worthwhile. In order to resolve some of the more serious problems it may be necessary to carry out additional measurements or structure refinements. However, the purpose of your study may justify the reported deviations and the more serious of these should normally be commented upon in the discussion or experimental section of a paper or in the "special_details" fields of the CIF. checkCIF was carefully designed to identify outliers and unusual parameters, but every test has its limitations and alerts that are not important in a particular case may appear. Conversely, the absence of alerts does not guarantee there are no aspects of the results needing attention. It is up to the individual to critically assess their own results and, if necessary, seek expert advice.

Publication of your CIF in IUCr journals

A basic structural check has been run on your CIF. These basic checks will be run on all CIFs submitted for publication in IUCr journals (*Acta Crystallographica*, *Journal of Applied Crystallography*, *Journal of Synchrotron Radiation*); however, if you intend to submit to *Acta Crystallographica Section C* or *E* or *IUCrData*, you should make sure that full publication checks are run on the final version of your CIF prior to submission.

Publication of your CIF in other journals

Please refer to the *Notes for Authors* of the relevant journal for any special instructions relating to CIF submission.

PLATON version of 02/02/2025; check.def file version of 02/02/2025

Datablock ky31 - ellipsoid plot

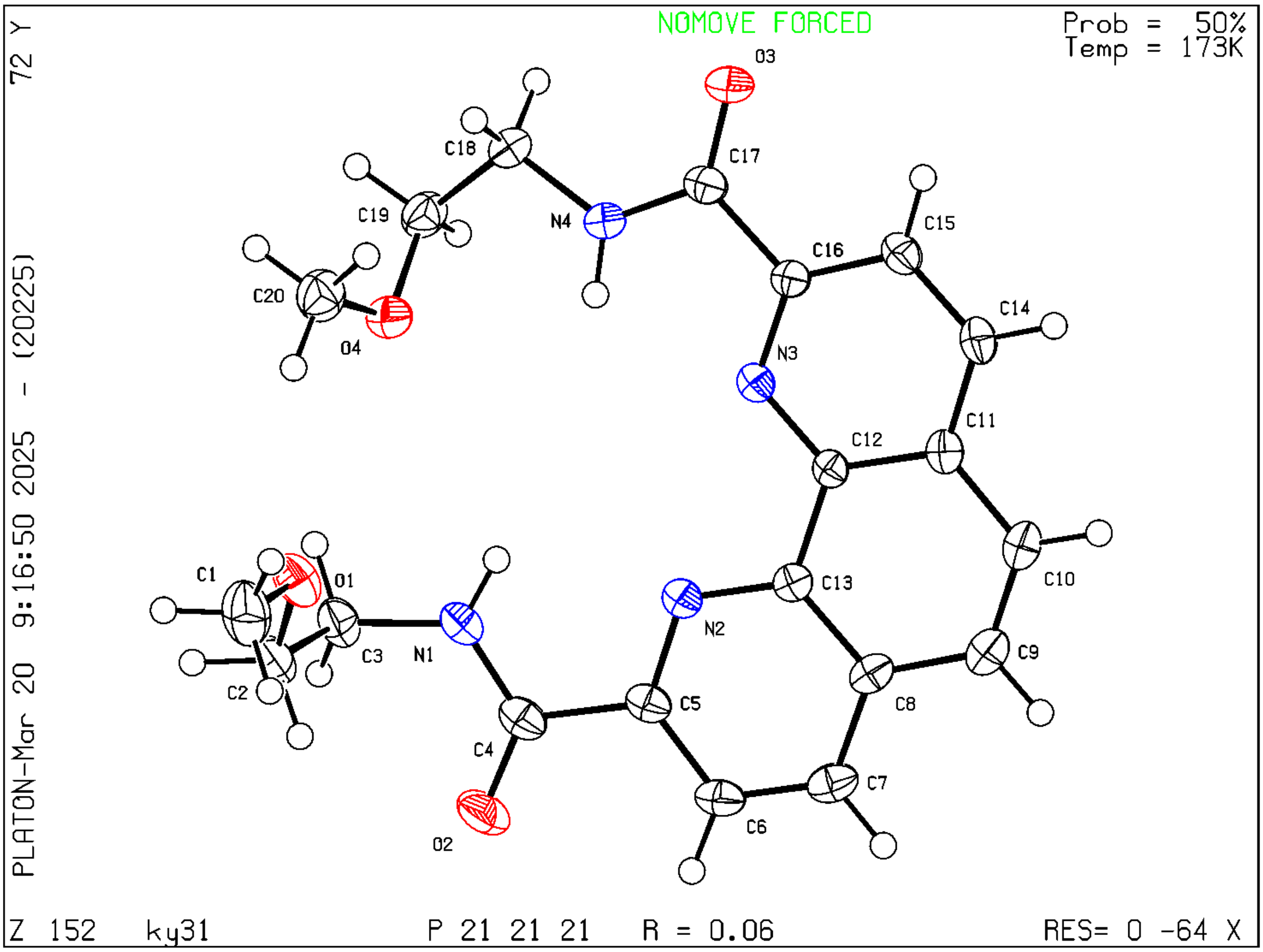

Download CIF editor (publCIF) from the IUCr
Download CIF editor (enCIFer) from the CCDC
Test a new CIF entry
