# Supplementary material for: On the Design of Effective Water‐Soluble Actinide‐Masking Ligands Through Ligand Structure Modulation
Source: Adv Sci (Weinh). 2025 Aug 21;12(42):e12292. doi: 10.1002/advs.202512292 (PMC12622539; doi:10.1002/advs.202512292)

checkCIF/PLATON report

Structure factors have been supplied for datablock(s) ccc_pl

THIS REPORT IS FOR GUIDANCE ONLY. IF USED AS PART OF A REVIEW PROCEDURE FOR PUBLICATION, IT SHOULD NOT REPLACE THE EXPERTISE OF AN EXPERIENCED CRYSTALLOGRAPHIC REFEREE.

No syntax errors found.   CIF dictionary   Interpreting this report

Datablock: ccc_pl

```
Bond precision:   C-C = 0.0084 A          Wavelength=0.71073

Cell:              a=15.6889(7)      b=18.8482(9)      c=8.7693(3)
                   alpha=90          beta=90           gamma=90
Temperature:       173 K
```

	Calculated	Reported
Volume	2593.15(19)	2593.15(19)
Space group	P n n 2	P n n 2
Hall group	P 2 -2n	P 2 -2n
Moiety formula	C20 H22 Eu N7 O13	C20 H22 Eu N7 O13
Sum formula	C20 H22 Eu N7 O13	C20 H22 Eu N7 O13
Mr	720.42	720.40
Dx,g cm-3	1.845	1.845
Z	4	4
Mu (mm-1)	2.499	2.499
F000	1432.0	1432.0
F000’	1432.08	
h,k,lmax	18,22,10	18,22,10
Nref	4802[2570]	4783
Tmin,Tmax	0.613,0.607	0.661,0.745
Tmin’	0.601	

```
Correction method= # Reported T Limits: Tmin=0.661 Tmax=0.745
AbsCorr = MULTI-SCAN

Data completeness= 1.86/1.00       Theta(max)= 25.422

R(reflections)= 0.0226( 4494)                    wR2(reflections)=
                                                 0.0515( 4783)
S = 1.064                  Npar= 373
```

The following ALERTS were generated. Each ALERT has the format
test-name_ALERT_alert-type_alert-level.
Click on the hyperlinks for more details of the test.

Alert level C

```
PLAT090_ALERT_3_C Poor Data / Parameter Ratio (Zmax > 18) ........       6.87 Note
PLAT220_ALERT_2_C NonSolvent   Resd 1  C   Ueq(max)/Ueq(min) Range        5.9 Ratio
PLAT220_ALERT_2_C NonSolvent   Resd 1  O   Ueq(max)/Ueq(min) Range        3.5 Ratio
PLAT222_ALERT_3_C NonSolvent Resd 1  H   Uiso(max)/Uiso(min) Range        6.2 Ratio
PLAT342_ALERT_3_C Low Bond Precision on  C-C Bonds ..............    0.00838 Ang.
```

Alert level G

```
PLAT002_ALERT_2_G Number of Distance or Angle Restraints on AtSite          3 Note
PLAT003_ALERT_2_G Number of Uiso or U(i,j) Restrained non-H Atoms           3 Report
PLAT007_ALERT_5_G Number of Unrefined Donor-H Atoms .............           2 Report
              H1    H4
PLAT176_ALERT_4_G The CIF-Embedded .res File Contains SADI Records          1 Report
PLAT186_ALERT_4_G The CIF-Embedded .res File Contains ISOR Records          1 Report
PLAT187_ALERT_4_G The CIF-Embedded .res File Contains RIGU Records          1 Report
PLAT860_ALERT_3_G Number of Least-Squares Restraints ............          41 Note
PLAT910_ALERT_3_G Missing # of FCF Reflection(s) Below Theta(Min).          2 Note
                1  1  0,   0  2  0,
PLAT912_ALERT_4_G Missing # of FCF Reflections Above STh/L=  0.600          6 Note
PLAT933_ALERT_2_G Number of HKL-OMIT Records in Embedded .res File          1 Note
               13 16  2,
PLAT969_ALERT_5_G The 'Henn et al.' R-Factor-gap value ..........       1.542 Note
              Predicted wR2: Based on SigI**2  3.34 or SHELX Weight  4.84
PLAT978_ALERT_2_G Number C-C Bonds with Positive Residual Density.          1 Info
```

0 **ALERT level A** = Most likely a serious problem - resolve or explain
0 **ALERT level B** = A potentially serious problem, consider carefully
5 **ALERT level C** = Check. Ensure it is not caused by an omission or oversight
12 **ALERT level G** = General information/check it is not something unexpected

0 ALERT type 1 CIF construction/syntax error, inconsistent or missing data
6 ALERT type 2 Indicator that the structure model may be wrong or deficient
5 ALERT type 3 Indicator that the structure quality may be low
4 ALERT type 4 Improvement, methodology, query or suggestion
2 ALERT type 5 Informative message, check

It is advisable to attempt to resolve as many as possible of the alerts in all categories. Often the minor alerts point to easily fixed oversights, errors and omissions in your CIF or refinement strategy, so attention to these fine details can be worthwhile. In order to resolve some of the more serious problems it may be necessary to carry out additional measurements or structure refinements. However, the purpose of your study may justify the reported deviations and the more serious of these should normally be commented upon in the discussion or experimental section of a paper or in the "special_details" fields of the CIF. checkCIF was carefully designed to identify outliers and unusual parameters, but every test has its limitations and alerts that are not important in a particular case may appear. Conversely, the absence of alerts does not guarantee there are no aspects of the results needing attention. It is up to the individual to critically assess their own results and, if necessary, seek expert advice.

Publication of your CIF in IUCr journals

A basic structural check has been run on your CIF. These basic checks will be run on all CIFs submitted for publication in IUCr journals (*Acta Crystallographica*, *Journal of Applied Crystallography*, *Journal of Synchrotron Radiation*); however, if you intend to submit to *Acta Crystallographica Section C* or *E* or *IUCrData*, you should make sure that full publication checks are run on the final version of your CIF prior to submission.

Publication of your CIF in other journals

Please refer to the *Notes for Authors* of the relevant journal for any special instructions relating to CIF submission.

PLATON version of 13/05/2024; check.def file version of 04/05/2024

Datablock ccc_pl - ellipsoid plot

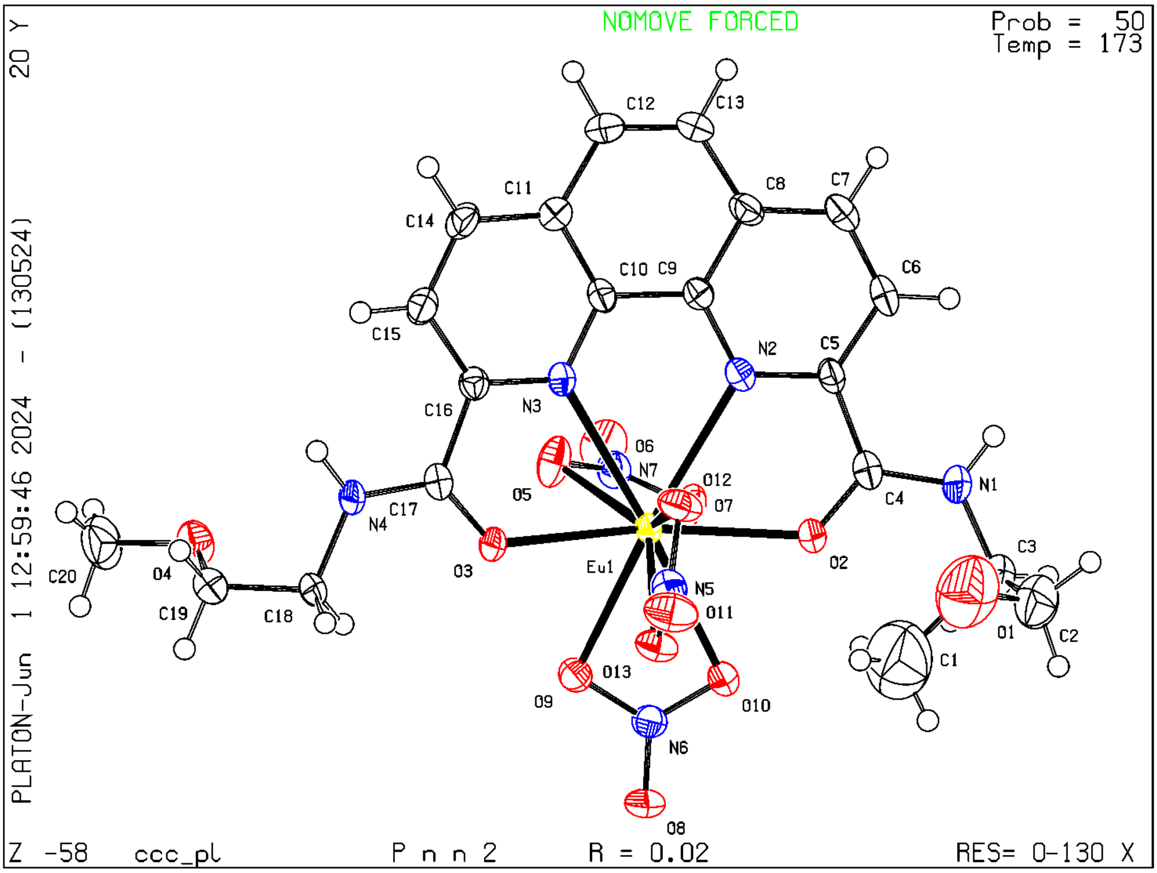

Supplement: Supplementary file 2 — Supporting Information [file ADVS-12-e12292-s001.zip › checkCIF_Phen-2DIC2OMe-Eu.pdf]
